# Tumor cell density dependent IL-8 secretion induces the fluctuation of tregs/CD8 + T cells infiltration in hepatocellular carcinoma: one prompt for the existence of density checkpoint

**DOI:** 10.1186/s12967-023-04060-3

**Published:** 2023-03-17

**Authors:** Mengchao Yan, Jia Yao, Yan Lin, Jun Yan, Ye Xie, Zongli Fu, Yongqiang Zhou, Jiayun Wei, Xun Li

**Affiliations:** 1 The First School of Clinical Medical, Lanzhou University, No.1 Donggang West Road, Chengguan District, Lanzhou, 730000 Gansu People’s Republic of China; 2 Key Laboratory of Biotherapy and Regenerative Medicine of Gansu Province, Lanzhou, 730000 Gansu People’s Republic of China; 3 Department of General Surgery, The First Hospital of Lanzhou University, Lanzhou, 730000 Gansu People’s Republic of China; 4 SUN YAT-SEN University, Guangzhou, 510000 Guangdong People’s Republic of China

**Keywords:** Tumor cell density, IL-8, Lactate, Density checkpoint

## Abstract

**Background:**

Tumor cell density is a basic pathological feature of solid tumors. Chemotherapy, radiotherapy, and targeted therapy reduce tumor cell density, whereas unrestricted tumor cell proliferation promotes this feature. The impact of tumor cells on the microenvironment following changes in tumor cell density is still unclear. In this study, we focused on the response of key immune cell subsets to tumor cell density in hepatocellular carcinoma (HCC).

**Methods:**

We determined the density of tumor and immune cells in the same area by section staining. We then identified potential mediators using polymerase chain reaction (PCR), enzyme-linked immunofluorescence assay (ELISA), 3D and co-culture, flow cytometry, and lentivirus intervention. The mechanism of lactate promotion was verified using lactate tests, bioinformatics, western blotting, and the above methods. The IL-8/DAPK1/lactate/regulatory T cell (Treg) axis was verified using a mouse liver cancer model. Tumor mutation burden was calculated using maftools in R.

**Results:**

We found that the Treg/CD8 + T cell ratio is not consistent with tumor cell density in HCC, and a decreased Treg/CD8 + T cell ratio in the range of 5000–6000 cells/mm^2^ may elicit the possibility for immunotherapy in an immunosuppressive microenvironment. We showed that IL-8 mediates this immune fluctuation and promotes the infiltration of Tregs through the DAPK1/pyruvate kinase activity/lactate axis in HCC. Based on tumor ploidy and mutation burden data, we discussed the potential significance of immune fluctuation in the homeostasis of HCC mutation burden and proposed a “density checkpoint” and “entropy model” to describe this phenomenon.

**Conclusions:**

In summary, we report the mode of infiltration of Tregs/CD8 + T cells in response to tumor cell density and provide a new theoretical basis for IL-8 as a therapeutic target and the selection of an immunotherapy window in HCC.

**Supplementary Information:**

The online version contains supplementary material available at 10.1186/s12967-023-04060-3.

## Background

Hepatocellular carcinoma (HCC) is the 6th most commonly diagnosed cancer and 3th leading cause of death globally [1]. The occurrence and development of HCC is a continuous process of population evolution. Tumor cell density refers to the number of tumor cells per unit volume or area, which responds to the proliferation of tumor cells, and its dynamic changes throughout the whole process of tumor development. Meanwhile, the secretome of tumor cells, such as cytokines and metabolites, can change greatly with the density of tumor cells and further drive the evolution of tumor cell behavior [2].

Lymphocytes are the main immune cells producing anti-tumor immunity, and their degree of infiltration has been previously evaluated to predict the clinical prognosis of HCC. The infiltration of CD8 + T cells in HCC is associated with better prognosis, while the presence of regulatory T cells (Tregs) is associated with poor prognosis [3, 4]. In addition, as an important part of the tumor immune microenvironment (TIME), tumor associated macrophages (TAMs) can polarize into two groups with opposite functions (M1 and M2). M1 macrophages play an anti-tumor role and M2 macrophages promote tumor progression and poor prognosis [5, 6]. The direction of polarization of M2/M1 macrophages can affect the prognosis of tumors [7]. Identifying influences on the polarization of Tregs/CD8 + T cells and M2/M1 macrophages can help us determine the effects of tumor related factors on the TIME.

In this study, by identifying and classifying infiltration data for CD8 + T cells, Tregs (FOXP3 +), and M1 (CD86 +) and M2 (CD206 +) macrophages in HCC tissues according to tumor cell density, we found that the ratio of Tregs/CD8 + T cells fluctuated with increasing tumor cell density, while the ratio of M2/M1 macrophages did not change significantly. We determined that the fluctuation of IL-8 expression in HCC was consistent with the fluctuation of the Treg/CD8 + T cell ratio and proved that IL-8 secreted by HCC enhanced the infiltration of Tregs by promoting lactate production. IL-8 promotes lactate production in HCC cells through the death-associated protein kinase 1 (DAPK1)/pyruvate kinase (PK) activity axis. In addition, we discussed the potential significance of immune fluctuations based on Hanlee P. Ji et al.’s theory and HCC tumor mutation burden (TMB) data, and proposed two concepts which may help explain the contradiction between the accumulation of genomic instability and tumor homeostasis: the “entropy model” and “density checkpoint”. In summary, in this study we describe the mode and mechanisms of infiltration of Treg/CD8 + T cell and their relation to tumor cell density. We further provide a new theoretical basis for IL-8 as a therapeutic target and its use for the selection of an HCC immunotherapy window.

## Methods

### HCC samples

HCC tissue sections from sixty-five cases were obtained from the Shanghai Outdo Biotech Company (Ethical approval number: SHYJS-CP-1707005). The clinical characteristics of these patients are displayed in Table 1.

**Table 1:** Baseline characteristics of the patients

	< 5000 cells/mm^2^ (n = 23)	5000–6000 cells/mm^2^ (n = 14)	> 6000 cells/mm^2^ (n = 28)	*P* value
Age (years)				0.515
Median	52	49	51	
Range	31–78	32–77	32–76	
Gender (m/f)	21/2	13/1	23/5	0.615
Number of lesions				1.000
1	21	13	25	
2	2	1	3	
Tumor size (cm)	4.8 ± 3.9	3.7 ± 2.5	4.4 ± 2.7	0.557
Pathologic grading (n)				0.857
I-II	2	1	1	
II	11	8	15	
II-III	4	3	8	
III	6	2	4	
TB (μmol/L)	16.3 ± 7.0	14.2 ± 5.8	13.8 ± 5.8	0.423
ALT (U/L)	58.1 ± 63.4	37.0 ± 23.2	52.0 ± 39.1	0.407
ALB (g/L)	43.7 ± 4.5	42.1 ± 5.9	43.4 ± 5.6	0.657
AFP (μg/L)	3160.7 ± 12,600.0	888.3 ± 1944.5	2781.3 ± 10,158.1	0.193
GGT (U/L)	87.6 ± 76.8	66.1 ± 64.9	77.2 ± 74.8	0.489
HBsAg + (n)	17	10	24	0.466

### Cell culture

The human HCC cell lines, Huh-7 and HCCLM3, were kindly provided by Cell Bank/Stem Cell Bank, Chinese Academy of Sciences, China. Hepa1-6 cells were obtained from Procell Life & Technology, China. Huh-7, HCCLM3, and Hepa1-6 cells were cultured in Dulbecco's modified eagle’s medium (DMEM, GIBCO, USA) supplemented with 10% fetal bovine serum (FBS, Hyclone Laboratories, USA) and 1% penicillin and streptomycin (Hyclone Laboratories, USA). The cells were maintained at 37 °C and 5% CO_2_ in a humidified incubator.

### 3D cell culture

HCC cells were collected and counted. The gradient number of HCC cells (4 × 10^5^, 8 × 10^5^, 12 × 10^5^, and 16 × 10^5^ cells) were centrifuged and resuspended with DMEM medium to a total volume of 20 µL. The cell suspension was mixed with 20 µL matrigel matrix (Corning Life Sciences, USA). After low-speed vibration, the mixed solution was placed in a 37 ℃ incubator for 15 min to promote the coagulation of cell clusters. Subsequently, cell clusters were transferred to a 6-well plate and cultured in complete medium for 24 h.

### Generation of knockdown and stably-expressed cell lines

HCCLM3, Huh-7, and Hepa1-6 cells were seeded in 6-well plates (Corning Life Sciences, USA). When the cell fusion rate reached 40–50%, target gene overexpression, target gene knockdown, and the corresponding negative control viruses (produced by Genechem, China) were transfected according to the manufacturer’s instructions. Sequences are shown in Additional file 1: Table S1. After 72 h of transfection, the transferred cells were continuously screened using purinomycin for 1 week to obtain stable cell lines; the screening concentration was 2 μg/mL. After screening, target gene expression was verified using enzyme linked immunosorbent assay (ELISA), quantitative polymerase chain reaction (qPCR), and western blot.

### ELISA

Gradient density HCC cells were seeded in 6-well plates (Corning Life Sciences, USA) and cultured for 24 h. The cell culture medium was centrifuged at 3000 g for 20 min. According to the manufacturer's instructions, a total of 100 μL of standard, control, and supernatant was added to each well of a 96-well plate. Biotinylated antibody (50 μL) was added to each well and incubated for 1 h at 20–25 ℃. Plates were washed three times with the wash solution, and 100 μL 1 × streptavidin-HRP solution was added into each well and incubated for 20 min at room temperature. After washing, 100 μL TMB was added into each well and incubated for 20 min at room temperature. Then, 100 μL stop solution was added. The absorbance was read at 450 nm after adding stop solution by microplate reader (BioTek, USA).

### Immunohistochemistry (IHC)

This experiment follows conventional methodology [8], which includes fixing fresh tissue, dehydrating, paraffin embedding, sectioning, mounting on slides, incubating with primary antibodies and corresponding secondary antibodies, and incubating with DAB Substrate (Solarbio, China). During this process, the sections were incubated with primary antibodies at 4 °C overnight. Color development was performed using DAB substrate for 60–90 s. Counterstaining was performed using hematoxylin. Antibodies used are shown in Additional file 1: Table S2. The images were acquired using a microscope (Leica, Germany). CD8 + T cells, Tregs, M1/M2 macrophages, and tumor cells were counted in five randomly selected high-power fields (200 ×).

### Co-culture of peripheral blood mononuclear cells (PBMCs) and HCC cells

Human blood PBMCs were isolated using PBMC separation medium (Solarbio, China). Isolated PBMCs were cultured in 1640 medium for 12 h to remove adherent cells. In the co-culture model, PBMCs (2 × 10^6^ cells/well) were inoculated into 6-well plates with 2.5 mL culture medium. HCC cells were inoculated into the 6-well Transwell inserts (Corning Life Sciences, USA) with 1.5 mL culture medium according to experimental conditions. PBMCs were allowed to co-culture with HCC cells for 24 h. After co-culture, the cells were washed with PBS three times and resuspended in 100 µl cell staining buffer (Biolegend, USA). Anti-mouse CD4-FITC (0.5 μL; Biolegend, USA) and anti-mouse CD25-APC (1 μL; Biolegend, USA) antibodies were added into each well and incubated at room temperature in the dark for 30 min. Subsequently, PBMCs were washed once with cell staining buffer. FOXP3 Fix/Perm solution (1 mL; Biolegend, USA) was added to each tube and incubated at room temperature in the dark for 20 min. PBMCs were washed once with cell staining buffer and resuspended in 1 mL FOXP3 Perm buffer at room temperature in the dark for 15 min. Then, PBMCs were centrifuged and resuspended in a solution of 100 µL FOXP3 Perm buffer and 2 μL anti-mouse Foxp3-PE antibody and incubated at room temperature in the dark for 30 min. Subsequently, cells were washed twice and resuspended in 0.5 mL cell staining buffer for analysis using a flow cytometer (Beckman, USA).

### Recombinant IL-8 intervention

The recombinant IL-8 (R&D, USA) was diluted to 10 µg/mL with sterile PBS. PBMCs (1 × 10^6^), 2 mL culture medium, and 20 ng IL-8 were added to each well in a six-well plate for incubation for 24 h. The proportion of PBMC subgroups was determined using flow cytometry.

### Lactate quantification

For determination of lactate levels, the cell culture medium was collected after 24 h of culture. After centrifugation at 3000 rpm for 10 min, the supernatant was collected for lactate quantification. For the determination of lactate levels in the tissue, each tissue sample (0.1 g) was homogenized with 200 µL PBS using an automatic homogenizer. After centrifugation, we obtained the supernatant and determined the lactate content using a lactate assay kit (Nanjing Jiancheng Bioengineering Institute, China) according to the manufacturer's instructions. A total of 20 μL of standard, control, and supernatant was added to each tube. One milliliter of the enzyme working solution and 200 μL chromogenic solution were added to each tube and incubated for 10 min at 37 ℃. Subsequently, 2 mL stop solution was added to each tube and the absorbance of 250 μL reaction solution was read at 530 nm. The supernatant protein content was determined for standardization of lactate expression.

### PK activity quantification

We cultured the cells in 6-well plates (Corning Life Sciences, USA) and washed them three times with PBS. PBS (100 µL) was freeze-thawed repeatedly, and we collected the supernatant after centrifugation. We determined PK activity using a pyruvate kinase assay kit (Nanjing Jiancheng Bioengineering Institute, China) according to the manufacturer’s instructions. The supernatant protein concentration was measured for standardization using an enhanced BCA protein assay kit (Beyotime Biotechnology, China).

### Extracellular acidification rate (ECAR)

A cell suspension (200 µL) was added to the 96-well plates and cultured in an incubator overnight until the cells covered the bottom. We then incubated the 96-well plates in a CO_2_-free incubator for 2 h before determination. An ECAR assay kit (Bestbio, China) was used to determine the extracellular acidification rate, according to the manufacturer's instructions. Cells were washed twice with the test buffer. Test buffer (90 μL) and acidified fluorescence probe (10 μL) were added to each well. A fluorescence microplate reader (Biotek, USA) was used to continuously detect the fluorescence signal.

### Western blot

We washed the cells cultured in 6-well plates (Corning Life Sciences, USA) three times in PBS and lysed them on ice in RIPA buffer, protease-inhibitor cocktail, and phosphatase-inhibitor (APExBIO, USA) solution. They were then mechanically broken down using a syringe. The suspension was centrifuged (4 ℃) and the supernatant was collected. An enhanced BCA protein assay kit (Beyotime Biotechnology, China) was used to determine the protein concentration of the supernatant. Proteins (50 μg) were separated on 10% SDS–polyacrylamide gels and transferred onto PVDF membranes (Millipore, USA). The PVDF membranes were blocked with 5% skim milk in TBS, then incubated with primary antibodies (overnight, 4 °C). After three washings in TBS-T, the membranes were incubated (25 °C, 1 h) with horse radish peroxidase-conjugated secondary antibodies (1:3000; Signalway Antibody, USA) and exposed to immobilon western chemilum hrp substrate (Millipore, USA). To confirm equal protein loading, we used anti-β-actin antibodies (1:2000, Signalway antibody, USA) to re-probe. The antibodies used are listed in Additional file 1: Table S2.

### Real-time quantitative reverse transcription PCR (qRT-PCR)

Total RNA was extracted from stomach tissues or cultured cells using Trizol solution (TAKARA, Japan). Two micrograms of total RNA was used for the reverse transcription reaction with a RevertAid First Strand cDNA Synthesis Kit (Thermo Fisher Scientific, USA). RT-qPCR was performed in a 20 µL final volume using 1 ng/µL cDNA and forward + reverse primers (500 nM each) as well as TB Green Premix Ex Taq II (TAKARA, Japan) in a CFX Connect PCR System (Bio-Rad, USA). The primer sequences are shown in Additional file 1: Table S3.

### Mouse HCC model

C57BL/6 mice (male, 18–20 g) were purchased from Vital River Laboratories (Beijing, China) and carefully maintained under standard laboratory conditions, and were acclimated for 7 d before the experiments and allowed free access to food and water. The mice were divided into four groups (n = 5). During the inoculation, the mice were placed under anesthesia on a sterile plate. The chests and abdomens were disinfected with iodophor. Next an opening approximately 1 cm below the abdominal white line was made and the liver lobe was gently fixed with a sterile cotton swab stained with PBS. We then inserted 5 × 10^6^ cells under the liver capsule. After hemostasis was achieved, the liver was gently pushed into the abdomen with a cotton swab, and the abdomen was closed layer by layer. The state of the mice was regularly observed. All animals received humane care according to the criteria outlined in the “Guide for the Care and Use of Laboratory Animals.” All animal tumor model schemes were approved by the Animal Care and Use Committee of the First Hospital of Lanzhou University (Ethics number: LDYYLL-2020-277).

### Analysis based on the cancer genome atlas (TCGA) database

We obtained RNAseq data for HCC from TCGA-LIHC dataset in TCGA database and calculated tumor cell density from hematoxylin & eosin (H&E)-stained sections (https://portal.gdc.cancer.gov/). Immune infiltration data for TCGA-LIHC dataset based on the CIBERSORT-ABS algorithm were obtained from the TIMER2.0 database (http://timer.cistrome.org/). The subclone expression and ploidy data for the HCC dataset in TCGA database were calculated using the CloneSig algorithm, and the data were obtained from the dataset provided by the author (reference 24). Single nucleotide variants and copy number variation data were downloaded from TCGA database, and TMB was calculated using maftools (version 2.8.5) in R (version 4.1.1).

### Statistical analysis

Data were analyzed using SPSS Statistics 25.0 (IBM, USA) and Prism 6.0 software (GraphPad Software, USA). Data are presented as the mean ± SD. The unpaired Student’s *t* tests or Mann–Whitney* U* test were used to compare the means of two groups. A one-way ANOVA or Kruskal–Wallis test were used for comparison among three or more groups. The chi-square test was used for statistics of classified data. Specific statistical methods were selected according to research purpose and normality and homogeneity of variance of the sample (Additional file 1: Table S4). Spearman’s test was used to analyze correlation. A *P* value of < 0.05 was considered statistically significant.

## Results

### The ratio of FOXP3 + /CD8 + fluctuates with increased tumor cell density

**Fig. 1:**
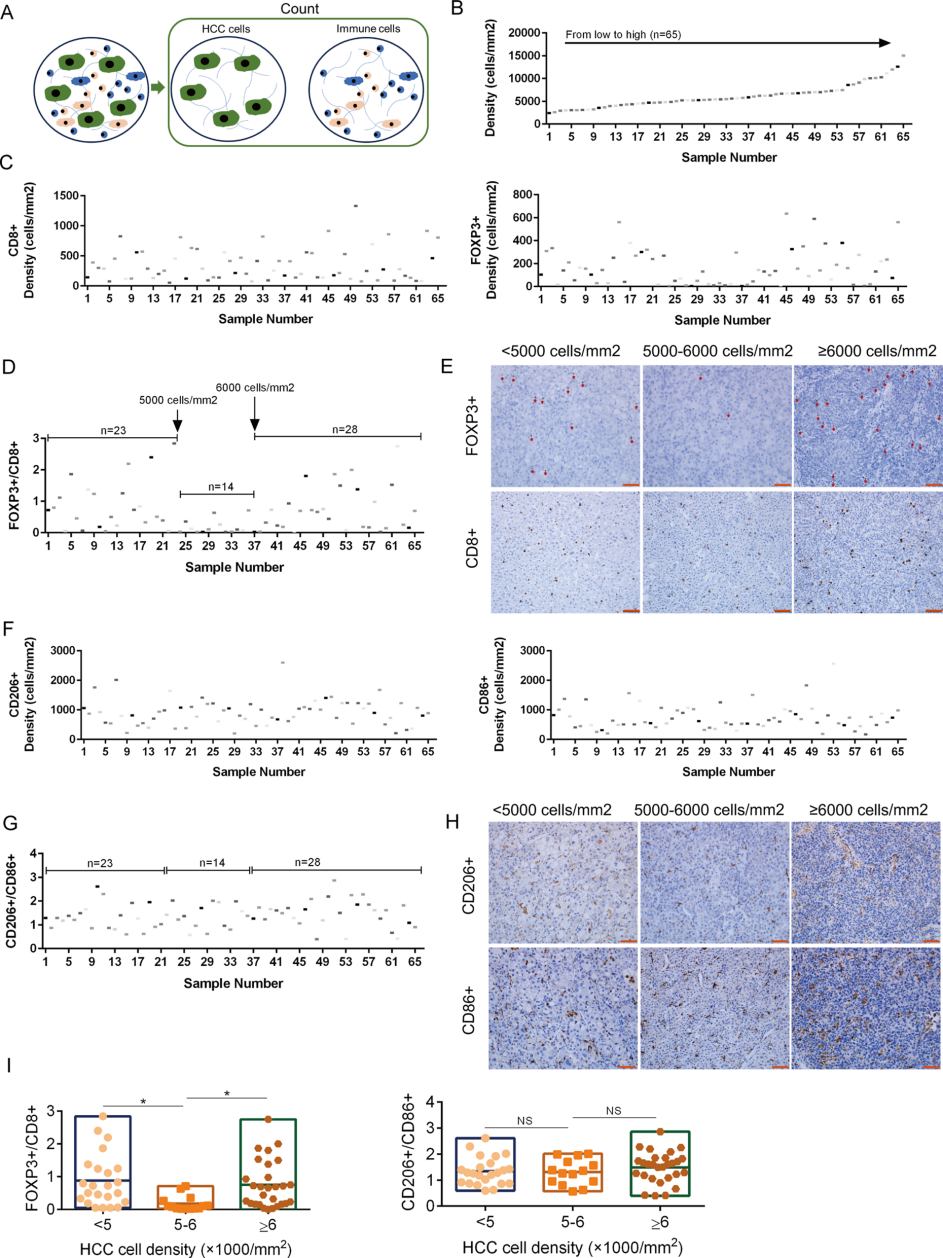
The change trend of FOXP3 + , CD8 + , CD206 + and CD86 + cells with tumor cell density. **A**. Counting tumor cell density and immune cell density in the same visual field.** B**. Quantification of tumor cell density (n = 65); Five visual fields were randomly selected for cell counting in each sample. **C**. Quantification of CD8 + and FOXP3 + cells density in HCC by immunohistochemistry (n = 65); Five visual fields were randomly selected for cell counting in each sample. **D**. Trends of FOXP3 + /CD8 + ratio with tumor cell density and grouping. **E**. FOXP3 + and CD8 + infiltration degree after grouping by HCC cell density (< 5000 cells/mm^2^, 5000–6000 cells/mm^2^ and ≥ 6000cells/mm^2^); Scale bar, 50 µm. **F**. Quantification of CD206 + and CD86 + cells density in HCC by immunohistochemistry (n = 65); Five visual fields were randomly selected for cell counting in each sample. **G**. Trends of CD206 + /CD86 + ratio with tumor cell density and grouping. **H**. CD206 + and CD86 + infiltration degree after grouping by HCC cell density (< 5000 cells/mm^2^, 5000–6000 cells/mm^2^ and ≥ 6000cells/mm^2^); Scale bar, 50 µm. **I**. Comparison of FOXP3 + /CD8 + ratio and CD206 + /CD86 + ratio between < 5000 cells/mm^2^ (n = 23), 5000–6000 cells/mm^2^ (n = 14) and ≥ 6000cells/mm^2^ (n = 28). The data represent the mean ± S.D. ANOVA or Kruskal–Wallis test were used for statistical analysis. *P < 0.05; **P < 0.01; ***P < 0.001

To analyze the distribution of Treg/CD8 + T cell infiltration on the whole tumor cell density axis, we quantified the degree of infiltration of CD8 + , FOXP3 + , CD86 + , and CD206 + cells in HCC samples (n = 65) by immunohistochemical staining [7]. The cell density of tumor tissues can be calculated by tissue staining [9, 10]. Tumor cells and immune cells were counted in the same field of vision (Fig. 1A). We arranged tumor cells according to density (low to high), and immune cell density was arranged according to tumor cell density (Fig. 1B–C). We observed that the FOXP3 + /CD8 + ratio fluctuated with tumor cell density (Fig. 1D–E). According to the fluctuation curve of the FOXP3 + /CD8 + ratio, we divided the samples into three groups (front stable section, valley section, and last stable section). The medium density group (5000–6000 cells/mm^2^) covered the FOXP3 + /CD8 + valley area, and the ratio was significantly different from that of the high and low density groups (Fig. 1I). The CD206 + /CD86 + ratio showed no significant difference among the three groups (Fig. 1F–I). There was no significant difference in age, sex, pathological grading, tumor size, serological index, or HBsAg among the three groups (Tab. 1). We further analyzed the correlation between the number of lesions, HBV infection, pathological grading, tumor size, and Treg/CD8 + T cell ratio, and we found no significant correlation between any of these variables (Additional file 1: Fig. S1A–D).

**Fig. 2:**
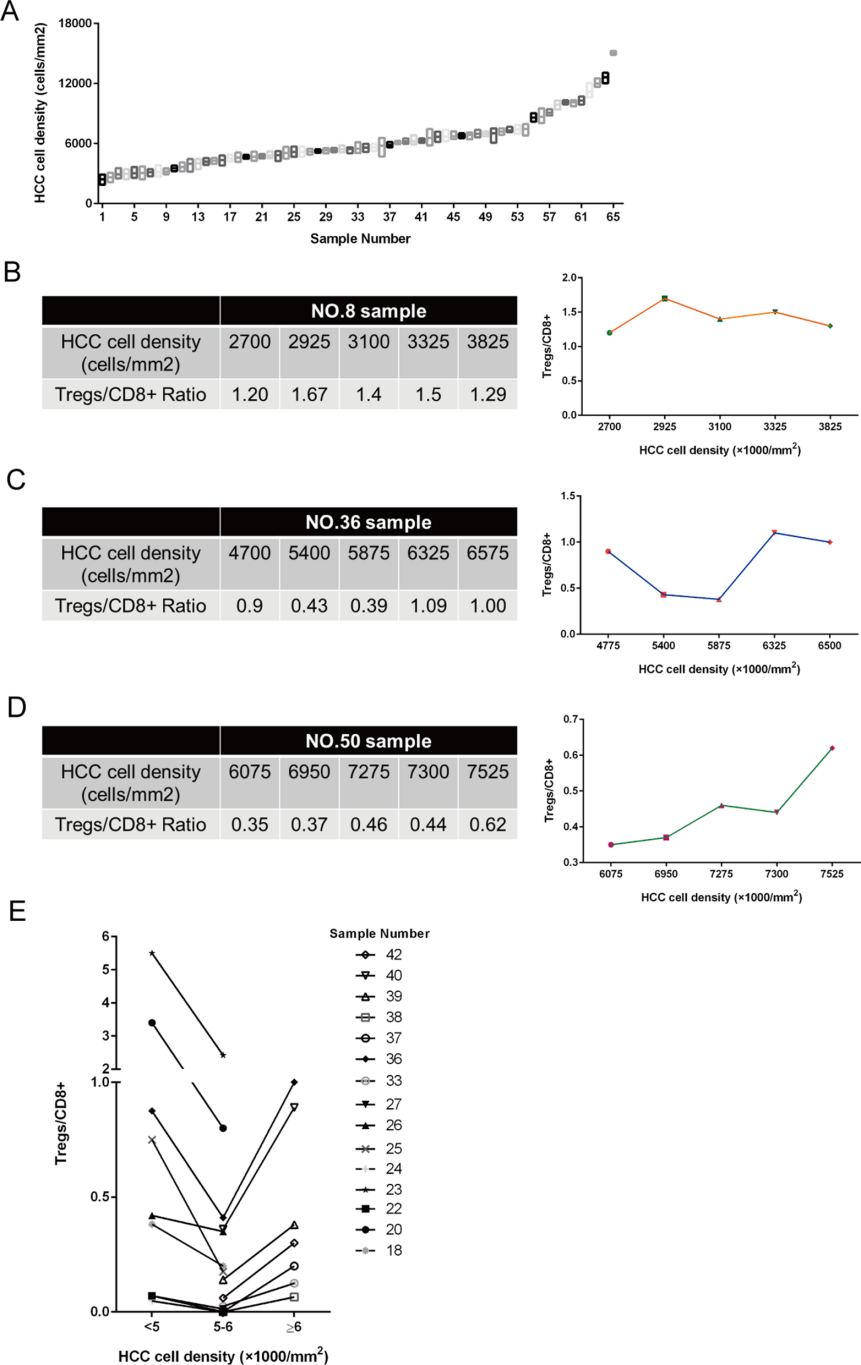
Tregs/CD8 + T cells fluctuation induced by regional heterogeneity of HCC. **A**. Heterogeneity of tumor cell density exists in different regions of the same HCC tissue; Five areas are selected in one sample; The data represent the mean ± S.D. **B**. In sample 8, Treg/CD8 + T cell ratio showed linear changes in different regions; Heterogeneous tumor cell density fluctuates within the range of < 5000 cells/mm^2^. **C**. In sample 36, Treg/CD8 + T cell ratio in different regions; Heterogeneous tumor cell density fluctuated across 5000–6000 cells/mm^2^. **D**. In sample 50, Treg/CD8 + T cell ratio showed linear changes in different regions; Heterogeneous tumor cell density fluctuated within the range of ≥ 6000 cells/mm^2^. **E**. Fifteen samples involving more than two intervals of < 5000 cells/mm^2^, 5000–6000 cells/mm^2^ and ≥ 6000 cells/mm^2^ were described for the change trend of Treg/CD8 + T cell ratio across intervals; Five areas were selected in one sample and Treg/CD8 + T cell ratio of the same interval was taken as an average value

Next, we analyzed the density heterogeneity in the same HCC sample (Fig. 2A). We randomly counted the tumor cell density and measured the degree of Treg/CD8 + T cell infiltration in 5 regions of the same sample. Due to the heterogeneity of tumor cell density in the same sample, we observed the effect of different densities on the infiltration of Treg/CD8 + T cell within the same sample. Typically, the Treg/CD8 + T cell ratio fluctuated when the heterogeneous density spanned the range of 5000–6000 cells/mm^2^ (Fig. 2B–D). In samples 8 (density < 5000) and 50 (density > 6000), Treg/CD8 + T cell infiltration showed linear changes. Due to the limited tissue area and heterogeneity, it is difficult to obtain a sufficient visual field for more than two density groups in one sample. After counting, we obtained 15 samples to analyze the trend in Treg/CD8 + T cell ratio when crossing two groups in the same sample, which is consistent with the fluctuation of the Treg/CD8 + T cell ratio we observed previously (Fig. 2E).

**Fig. 3:**
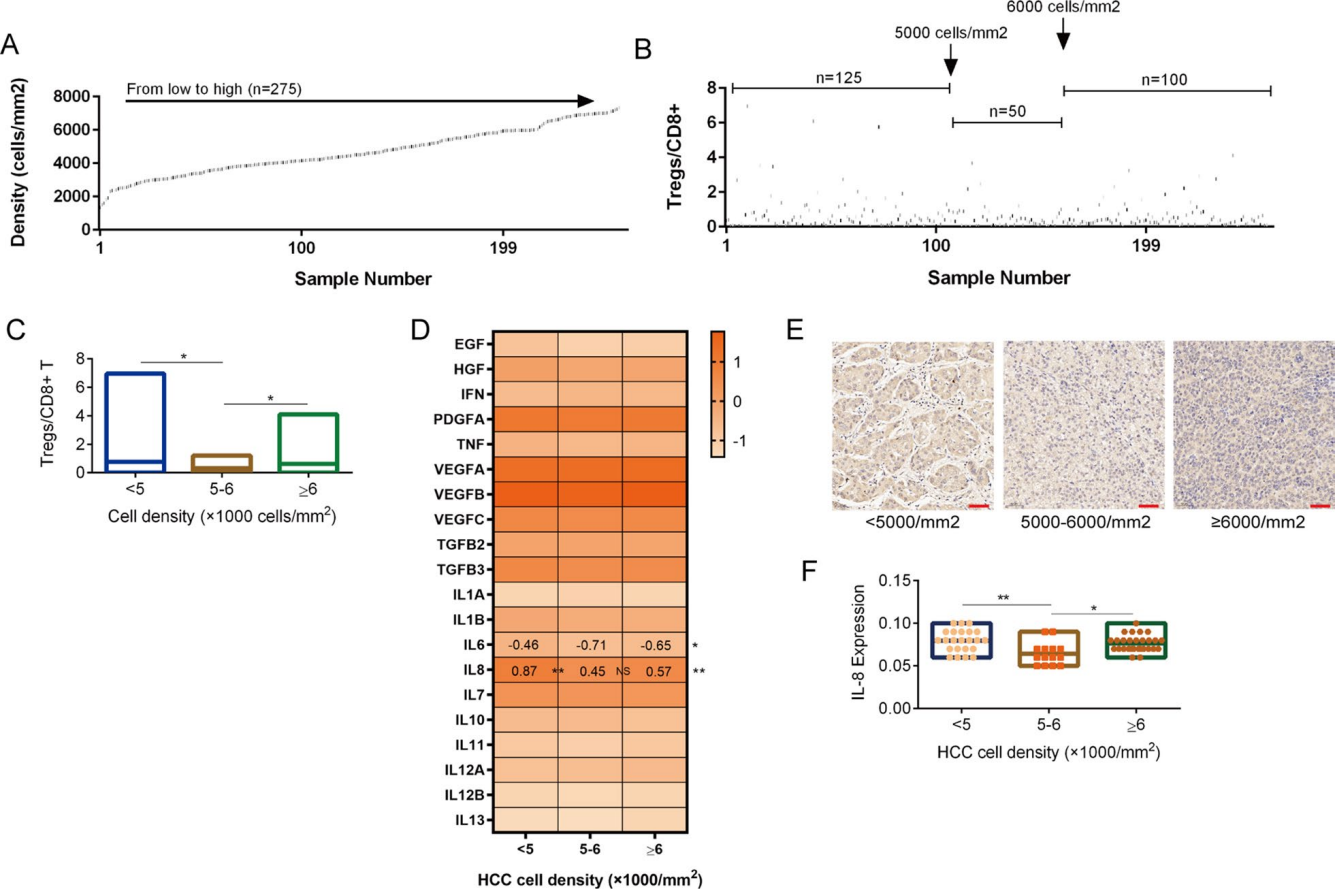
The fluctuation of IL-8 expression on the tumor cell density axis is consistent with Tregs/CD8 + T cell infiltration. **A**. Quantification of tumor cell density in HCC dataset of TCGA database; Five visual fields were randomly selected for cell counting in each sample. **B**. Ratio of infiltration degree of Treg and CD8 + T cell after grouping by HCC cell density (< 5000 cells/mm^2^, 5000–6000 cells/mm^2^ and ≥ 6000cells/mm^2^); Data obtained from the TCGA database; Quantification of infiltration degree based on CIBERSORT-ABS algorithm. **C**. Trends of FOXP3 + /CD8 + ratio with tumor cell density and grouping. **D**. mRNA expression of soluble factors in HCC after grouping by HCC cell density (< 5000 cells/mm^2^, 5000–6000 cells/mm^2^ and ≥ 6000cells/mm^2^); The number shows the expression of factors with significant difference. Data obtained from the TCGA database. **E**. The protein expression of IL-8 in HCC after grouping by HCC cell density (< 5000 cells/mm^2^, 5000–6000 cells/mm^2^ and ≥ 6000cells/mm^2^); Scale bar, 50 µm. **F**. Quantification of IL-8 protein expression in HCC after grouping by HCC cell density (< 5000 cells/mm^2^, 5000–6000 cells/mm^2^ and ≥ 6000cells/mm^2^) (n = 65); Image J was used for quantification. The data represent the mean ± S.D. ANOVA or Kruskal–Wallis test were used for statistical analysis. *P < 0.05; **P < 0.01; ***P < 0.001

In addition, we used the HCC dataset from TCGA database as the validation set and verified the association between tumor cell density and immune fluctuation. We obtained H&E staining data of HCC samples from TCGA database and calculated the cell density (Fig. 3A). Treg and CD8 + T cell infiltration data (CIBERSORT-ABS algorithm) were divided into three groups according to HCC cell density, and the Treg/CD8 + T cell ratio was found to decrease significantly in the medium density group (5000–6000 cells/mm^2^) (Fig. 3B, C). We used RNAseq data from TCGA database to screen the potential mediator of this phenomenon. The secretome is the most direct way for tumor cells to affect the TIME, and we first screened soluble factors to identify potential mediators. We matched cell density data with mRNA expression data for twenty main soluble factors and divided the samples into three groups based on cell density and the above rule. We observed that IL-8 was the only factor with the same fluctuation trend (Fig. 3D). Next, HCC samples (n = 65) were used to verify the expression of IL-8 by immunohistochemistry. We quantified IL-8 expression with ImageJ software, and the same trend was observed between the expression of IL-8 protein and the ratio of Tregs/CD8 + T cells (Fig. 3E–F).

### IL-8 secreted by HCC mediates immune fluctuation

**Fig. 4:**
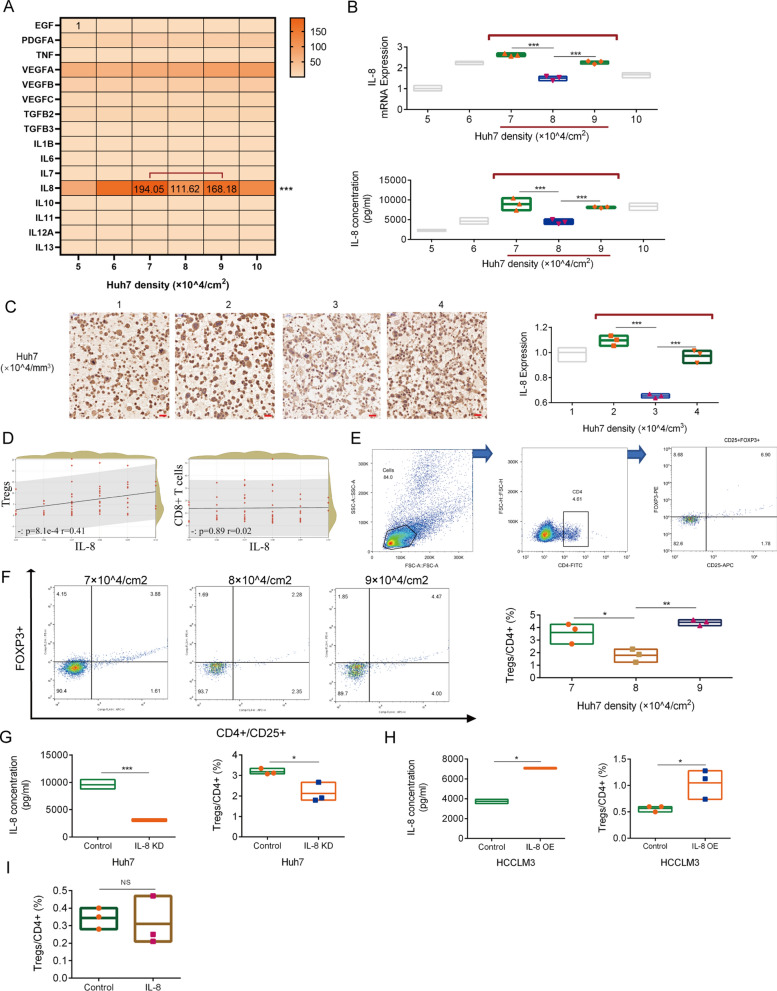
IL-8 is the mediator of density related immune fluctuations. **A**. mRNA expression of soluble factors in Huh-7 cells with different cell densities; determination after 24 h of culture. **B**. IL-8 mRNA expression of Huh-7 cells at different cell densities, and IL-8 concentration of Huh-7 cells in culture medium of different cell densities; determination after 24 h of culture. **C.** Quantification of IL-8 protein expression in the 3D culture of Huh-7 cells with different cell densities; Image J was used for quantification; Three visual fields were randomly selected for counting in each sample; scale bar, 50 µm. **D**. Spearman correlation scatter plot of IL-8 expression and lymphocytes including Tregs and CD8 + T cells in HCC (n = 65). **E**. Gating strategy of flow cytometry analysis. **F**. Effect of coculture with gradient density Huh7 with IL-8 fluctuation on Tregs polarization in PBMCs; determination after 24 h of culture. **G**. Effect of coculture with IL-8 KD and control Huh-7 cells (1 × 10^5^ cells/cm^2^) on Tregs polarization in PBMCs; IL-8 concentration in the culture medium of IL-8 KD and control Huh-7 cells (1 × 10^5^ cells/cm^2^); determination after 24 h of culture. **H**. Effect of coculture with IL-8 OE and control HCCLM3 cells (2 × 10^5^ cells/cm^2^) on Tregs polarization in PBMCs; IL-8 concentration in the culture medium of IL-8 OE and control HCCLM3 cells (2 × 10^5^ cells/cm^2^); determination after 24 h of culture. **I**. Effect of recombinant IL-8 (10 ng/mL, 24 h) on Tregs polarization in PBMCs; determination after 24 h of culture. The data represent the mean ± S.D. of three independent experiments. Mann–Whitney U test or t-tests were used to compare the means of two groups. ANOVA were used for comparison among three groups. *P < 0.05; **P < 0.01; ***P < 0.001. Abbreviations: KD, knockdown; OE, overexpression; PBMCs, peripheral blood mononuclear cells

We simulated gradient HCC cell density in vitro. mRNA expression of 16 soluble factors was quantified with tumor cell density, and we found that IL-8 was the only factor with fluctuations (HGF, IL-1A, and IL-12B expression levels were extremely low) (Fig. 4A). The mRNA expression and medium concentration of IL-8 fluctuated with cell density, especially in the range of 7–9 × 10^4^/cm^2^ cells (Fig. 4B). In addition, we observed HCC tissue as a three-dimensional structure and consequently established a three-dimensional culture system with gradient HCC cell densities using a matrix. Huh-7 cells in our 3D culture showed consistent fluctuation in IL-8 expression at 2–4 × 10^4^/cm^2^ cells (Fig. 4C). Next, immune infiltration analysis based on 65 HCC samples showed that IL-8 promote Treg infiltration (Fig. 4D). Therefore, we considered that the fluctuation of IL-8 further mediates the fluctuation of Treg/CD8 + T cell infiltration.

The residence of Tregs in tumors is regulated by a variety of factors including nutrition, metabolites, and cytokines [11]. As mentioned previously, we found that the expression of IL-8 is positively correlated with the content of Tregs in HCC. In order to investigate the mechanism of association between IL-8 and Tregs, we simulated the interaction in the microenvironment by co-culture in vitro and observed the responsiveness of Tregs to microenvironment changes by measuring Treg polarization. To verify the effect of gradient HCC cell density on Treg polarization, we co-cultured human PBMCs with HCC cells with gradient density (7–9 × 10^4^/cm^2^ cells) and measured the polarization of Tregs in CD4 + T cells (Fig. 4E). The percentage of Tregs in our co-culture system consistently fluctuations with that of those in HCC samples (Fig. 4F). Next, we established the Huh-7 cell line with low IL-8 expression based on shRNA (Fig. 4G). After co-culture, the percentage of Tregs in the control group was significantly higher than that in the IL-8 knockdown group (Fig. 4G). Next, we used another HCC cell line with IL-8 low expression (HCCLM3) and its IL-8 overexpression cell line to verify this conclusion and found the percentage of Tregs in the IL-8 overexpression group was significantly higher than that in the control group (Fig. 4H). However, when we directly challenged PBMCs with IL-8, there was no change in Treg polarization (Fig. 4I). Therefore, we can draw the following conclusions: First, gradient density HCC cells induce Treg polarization fluctuations in vitro. Second, IL-8 mediates the interactions between HCC cells and Treg polarization. Third, IL-8 promotes Treg polarization through indirect action.

### IL-8 promotes lactate production in HCC cells

**Fig. 5:**
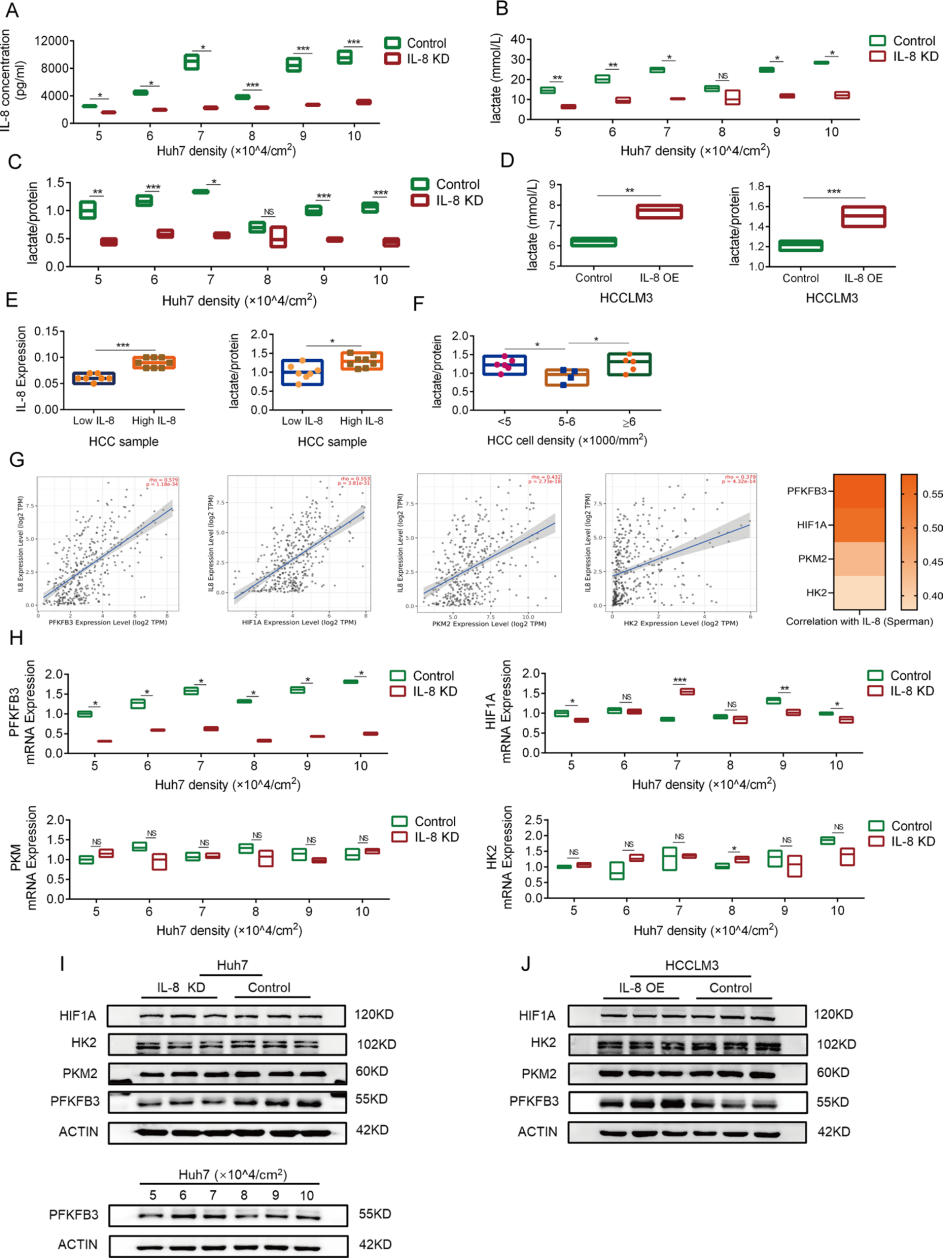
IL-8 enhances lactate production of HCC cells. **A**. IL-8 concentration in the culture medium of IL-8 KD and control Huh-7 cells; determination after 24 h of culture. **B**. Quantification of Huh-7 lactate production in the IL-8 KD and control groups; determination after 24 h of culture. **C**. Quantification of Huh-7 lactate production in the IL-8 KD and control groups; the protein concentration was used to standardize lactate production; determination after 24 h of culture. **D**. Quantification of HCCLM3 (2 × 10^5^ cells/cm^2^) lactate production in the IL-8 OE and control groups; the protein concentration was used to standardize lactate production; determination after 24 h of culture. **E**. Expression of lactate in HCC samples in low IL-8 group (n = 7) and high IL-8 group (n = 8); IL-8 expression data obtained from IHC quantified by Image J. **F**. Comparison of lactate expression between < 5000 cells/mm^2^ (n = 6), 5000–6000 cells/mm^2^ (n = 4) and ≥ 6000cells/mm^2^ (n = 5) in HCC samples. **G**. Spearman correlation scatter plot of IL-8 and four key enzymes mRNA expression in HCC; Analysis using TCGA database. **H**. mRNA expression of PFKFB3, HIF1A, PKM2, and HK2 in IL-8 KD and control Huh-7 cells of different cell densities. **I**. The protein expression of PFKFB3, HIF1A, PKM2, and HK2 in IL-8 KD and control Huh-7 cells; the protein expression of PFKFB3 in Huh-7 cells with different cell densities. **J**. The protein expression of PFKFB3, HIF1A, PKM2, and HK2 in IL-8 OE and control HCCLM3 cells. The data represent the mean ± S.D. of three independent experiments. Mann–Whitney U test or t–tests were used to compare the means of two groups. ANOVA were used for comparison among three groups. *P < 0.05; **P < 0.01; ***P < 0.001. Abbreviations: KD, knockdown; OE, overexpression

The IL-8 overexpressed Huh-7 cell culture medium was acidified in a shorter time. To explore the effect of IL-8 on HCC cell lactate production, we determined the lactate concentration of IL-8 knockdown Huh-7 and control cells in the culture medium and found that IL-8 promoted the production of lactate (Fig. 5A–C). This was verified by observing the IL-8 overexpressed HCCLM3 and control cells (Fig. 5D). Next, we obtained 15 frozen samples of HCC cells to determine lactate expression. According to the matched IL-8 IHC result, they were divided into two groups (Fig. 5E). The lactate expression of cells in the IL-8 overexpressed group was significantly higher than that of cells in the low expression group (Fig. 5E). Differences in lactate expression also existed in different tumor cell density groups (Fig. 5F). Lactate is a key metabolite in Treg-related immunosuppression and many studies have confirmed that a high lactate environment promotes the residence of Tregs in tumors and impairs the infiltration of CD8 + T cells [12–14]. Promoting lactate production may be the key mechanism of IL-8 in promoting an increased Treg/CD8 + T cell ratio. Blocking the pro-lactate effect of IL-8 is necessary in determining the effect of lactate. First, we explored the mechanism of increasing lactate production.

Key glycolytic enzymes are the core of lactate production regulation in tumor cells [15]. We analyzed the mRNA expression correlation between IL-8 and the key glycolytic enzymes involved in HCC using the cBioPortal database to analyze the HCC dataset in TCGA. The three most relevant were 6-phosphofructo-2-kinase/fructose-2,6-biphosphatase 3 (PFKFB3), pyruvate kinase M2 (PKM2), and hexokinase 2 (HK2) and hypoxia inducible factor 1 subunit alpha (HIF1A) (Fig. 5G). We quantified the mRNA and protein expressions of the enzymes in established Huh-7 cells, and only PFKFB3 showed significant correlation with IL-8 (Fig. 5H–I). We also verified the promoting effects of IL-8 on PFKFB3 expression in HCCLM3 cells (Fig. 5J).

**Fig. 6:**
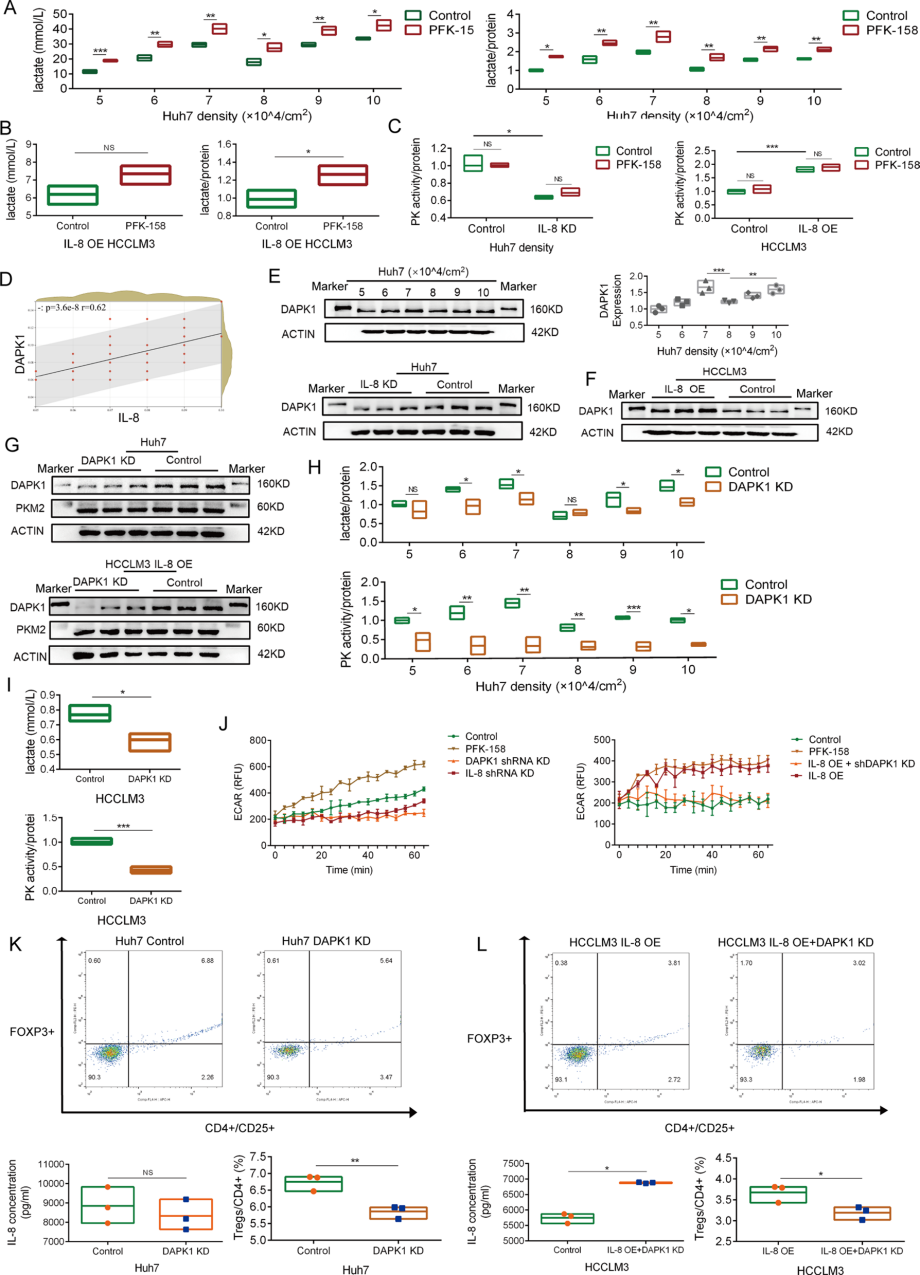
Enhanced lactate production is mediated by IL-8/DAPK1/PK axis. **A**. Quantification of Huh-7 lactate production in PFK-158 and control group; determination after 24 h of culture; the protein concentration was used to standardize lactate production. **B**. Quantification of HCCLM3 (2 × 10^5^ cells/cm^2^) lactate production in PFK-158 and control group; determination after 24 h of culture; the protein concentration was used to standardize lactate production. **C**. Quantification of Huh-7 and HCCLM3 PK activity; control and IL-8 KD or OE HCC cells were intervened with PFK-158 (10 µM for 24 h); the protein concentration was used to standardize PK activities. **D**. Spearman correlation between IL-8 and DAPK1 expression in HCC; IHC and Image J were used for quantification; n = 65. **E**. The protein expression of DAPK1 in the IL-8 KD and control Huh-7 cells; The protein expression of DAPK1 in Huh-7 cells with different cell densities. **F**. The protein expression of DAPK1 in IL-8 OE and control HCCLM3 cells. **G**. The protein expressions of DAPK1 and PKM2 in DAPK1 KD and control Huh-7/HCCLM3 IL-8 OE cells. **H**. Quantification of lactate production and PK activity in DAPK1 KD and control Huh-7 cells of different cell densities; determination after 24 h of culture. **I**. Quantification of lactate production and PK activity in DAPK1 KD and control HCCLM3 IL-8 OE cells (2 × 10^5^ cells/cm^2^) of different cell densities; determination after 24 h of culture. **J.** Quantification of ECAR in Huh-7 and HCCLM3 cells with PFK-158, DAPK1 KD, and IL-8 KD/OE. **K**. Effect of coculture with DAPK1 KD and control Huh-7 cells (1 × 10^5^ cells/cm^2^) on Tregs polarization in PBMCs; IL-8 expression of DAPK1 KD and control Huh-7 cells (1 × 10^5^ cells/cm^2^); determination after 24 h of culture. **L**. Effect of coculture with DAPK1 KD and control IL-8 OE HCCLM3 cells (2 × 10^5^ cells/cm^2^) on Tregs polarization in PBMCs; IL-8 expression of DAPK1 KD and control IL-8 OE HCCLM3 cells (2 × 10^5^ cells/cm^2^); determination after 24 h of culture. The data represent the mean ± S.D. of three independent experiments. Mann–Whitney U test or t-tests were used to compare the means of two groups. ANOVA were used for comparison among three groups. *P < 0.05; **P < 0.01; ***P < 0.001. Abbreviations: KD, knockdown; OE, overexpression; PBMCs, peripheral blood mononuclear cells; PK, pyruvate kinase

PFKFB3 is a bifunctional enzyme with kinase and phosphatase activity which controls the flow of glycolysis [16]. PFK-158 is a highly specific PFKFB3 inhibitor and is currently under evaluation in a Phase I trial (ClinicalTrials.gov identifier: NCT02044861). Next, we used PFK-158 to inhibit PFKFB3 activity, and found that PFK-158 promoted lactate production in Huh-7 and HCCLM3 cells (Fig. 6A, B). Enhanced lactate production was not induced by high expression of PFKFB3.

### IL-8 enhances PK activity by promoting DAPK1 expression

We measured PK activity and found that high PFKFB3 expression did not significantly change the PK activity in HCC; however cells in the IL-8 overexpressed group showed higher PK activity (Fig. 6C). We considered that the increase in PK activity was an important cause of the increase in lactate production in the IL-8 overexpression group. PK transfers a high-energy phosphate group from phosphoenolpyruvate to ADP to form pyruvate, which participates in the final reaction of glycolysis. PKM2, a member of the PK family, is upregulated in most tumors and necessary for aerobic glycolysis in tumors [17]. PKM2 is highly adaptable and can coordinate with the multiple metabolic changes of tumors [17–19]. High PFKFB3 expression induced by IL-8 did not enhance PK activity in HCC. Therefore, we considered that PK activity was promoted through other pathways.

Metabolism-related molecules are important participants in the regulation of PKM2 activity, including serine and succinylaminoimidazolecarboxamide ribose-5′-phosphate (SAICAR) [20, 21]. Significant changes in the extracellular nutritional environment are necessary for serine and SAICAR to regulate PKM2 activity in stabilizing cell proliferation. In our cell cultures, we used a complete medium supplemented with serine and sufficient glucose to provide appropriate nutrition to find a non-metabolic pathway that allowed for the connection between IL-8 and PKM2 activity. This allowed IL-8 to support lactate production stably in complex nutritional states.

DAPK1, as a tumor suppressor protein, has been reported to directly bind and functionally activate PKM2 [22]. We used the TIMER database to visualize the correlation between IL-8 and DAPK1 in HCC. There was a positive correlation between IL-8 and DAPK1 (Additional file 1: Fig. S2A). We speculated that DAPK1 expression was enhanced by IL-8, which promoted PK activity. Next, we quantified DAPK1 expression in HCC by IHC and matched it with IL-8 expression. Spearman correlation analysis showed that DAPK1 was positively correlated with IL-8 expression and the expression of DAPK1 also fluctuated with tumor cell density (Fig. 6D and Additional file 1: Fig. S2B). In vitro, we quantified the expression of DAPK1 in Huh-7 and HCCLM3 cells and found that IL-8 enhanced DAPK1 expression (Fig. 6E, F). To verify the role of DAPK1 in the enhanced PK activity, we established Huh-7 and HCCLM3 cell lines with DAPK1 knockdown (Fig. 6G). DAPK1 knockdown did not induce changes in PKM2 expression (Fig. 6G). In DAPK1 knockdown cells, lactate production and PK activity decreased with no change in PKM2 expression (Fig. 6 H, I). Therefore, IL-8 promoted PK activity and lactate production by enhancing the expression of DAPK1 rather than PFKFB3. Analysis of ECAR data also verified this conclusion (Fig. 6J).

Next, HCC cells with different levels of lactate production were co-cultured with PBMCs to verify the role of lactate production in IL-8-induced Treg polarization. We found that the degree of Treg polarization in cells in the high lactate expression group was significantly higher than that of the low lactate expression group and that this change was not related to IL-8 expression (Fig. 6K–L). Therefore, IL-8 induces Treg polarization by promoting the production of lactate in HCC cells.

### Lactate is the key factor in IL-8 promoting treg polarization in vivo

**Fig. 7:**
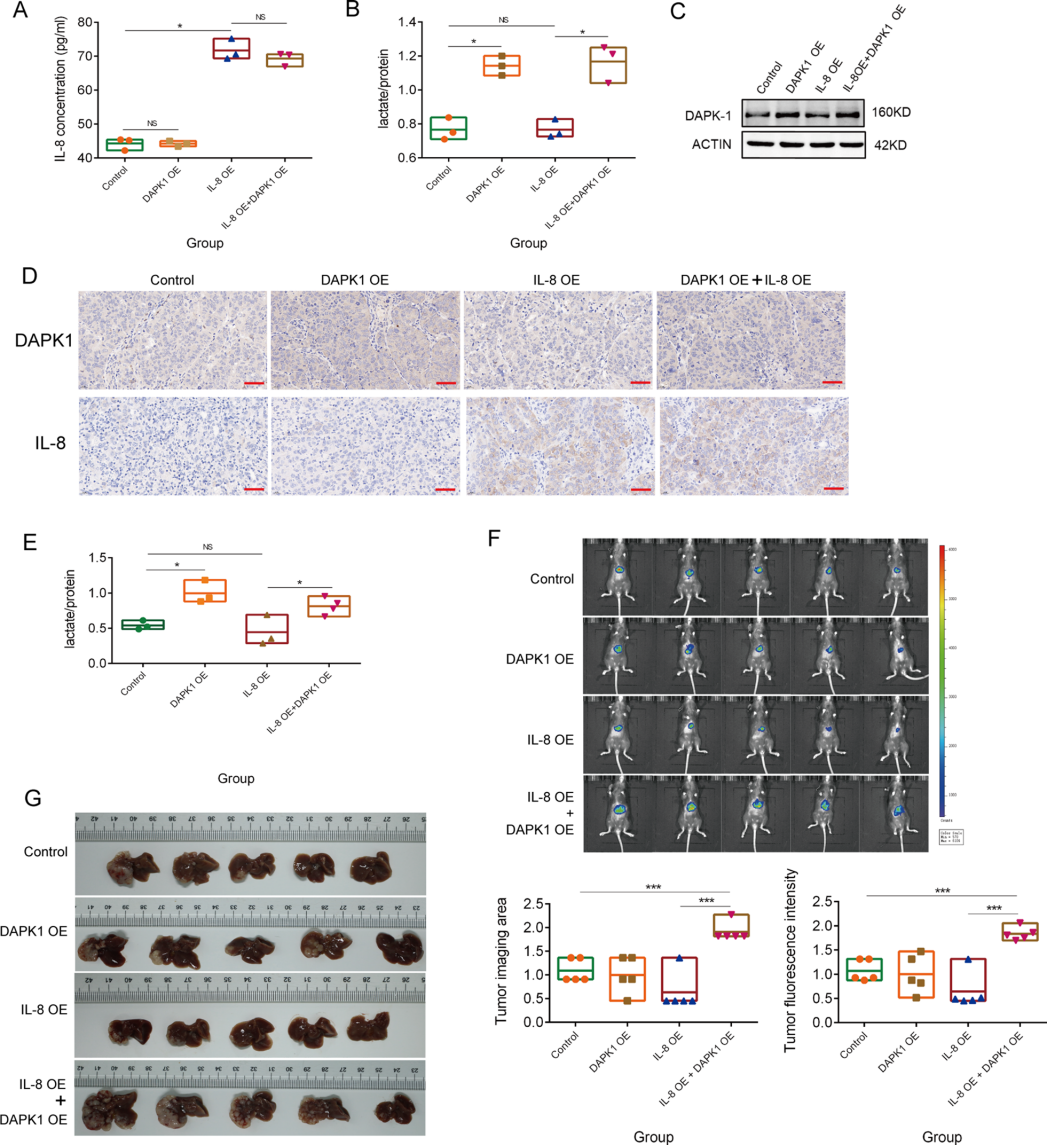
IL-8 combined with high lactate promotes the development of HCC. **A**. Quantification of IL-8 expression in the four groups including DAPK1 OE, IL-8 OE, IL-8 OE with DAPK1 OE, and control Hepa1-6 groups; the number of cells is 1 × 10^5^; determination after 24 h of culture. **B**. Quantification of lactate production in four groups of Hepa1-6 cells; determination after 24 h of culture. **C**. The protein expression of DAPK1 in four groups of Hepa1-6 cells. **D**. The expression of IL-8 and DAPK1 in the four mice orthotopic HCC groups; Immunohistochemistry was used to determine IL-8 and DAPK1 protein expressions; Scale bar, 50 µm. **E**. Quantification of lactate concentration in tumor tissues of the four mouse orthotopic HCC groups (n ≥ 3). **F**. In vivo fluorescence images of the four mouse orthotopic HCC groups (n = 5); Three weeks after Hepa1-6 implantation. **G**. Macroscopic tumor growth in the four groups orthotopic HCC model. The data represent the mean ± S.D. Mann–Whitney U test or t–tests were used for statistical analysis. *P < 0.05; **P < 0.01; ***P < 0.001. Abbreviations: KD, knockdown; OE, overexpression

In this section, we validated the role of lactate in IL-8 promoting Treg polarization in vivo. The degree of polarization of Tregs and CD8 + T cells were the main indicators. Mouse hepatoma cells, Hepa1-6 cells, were transplanted to establish an orthotopic HCC model. We overexpressed IL-8 in Hepa1-6 cells and found no change in lactate production (Fig. 7A, B). Subsequently, we quantified the protein expression of DAPK1 and found that DAPK1 expression was not enhanced by IL-8 in Hepa1-6 cells (Fig. 7C). This may be due to species differences. IL-8 is absent from the mouse genome, but mouse CXCR1 can bind IL-8 with reasonable affinity and produce functional responses [23]. This functional response may not be exactly the same as in human cells. To mimic the phenotype of human HCC cells, we overexpressed DAPK1 in Hepa1-6 cells (Fig. 7C). Enhanced DAPK1 expression promoted lactate production in Hepa1-6 cells (Fig. 7B). Thus, we established four models, including control cells, a high lactate group (DAPK1 overexpression, DAPK1 OE), an IL-8 overexpression group (IL-8 OE), and an IL-8 overexpression with high lactate group (IL-8 OE + DAPK1 OE) (Fig. 7D, E). Three weeks after Hepa1-6 implantation, the size of the HCC was observed through in vivo fluorescence imaging and anatomical observation (Fig. 7F, G). The IL-8 OE + DAPK1 OE group displayed the largest tumor size. These results suggest that lactate is crucial in the tumor-promoting effects of IL-8.

**Fig. 8:**
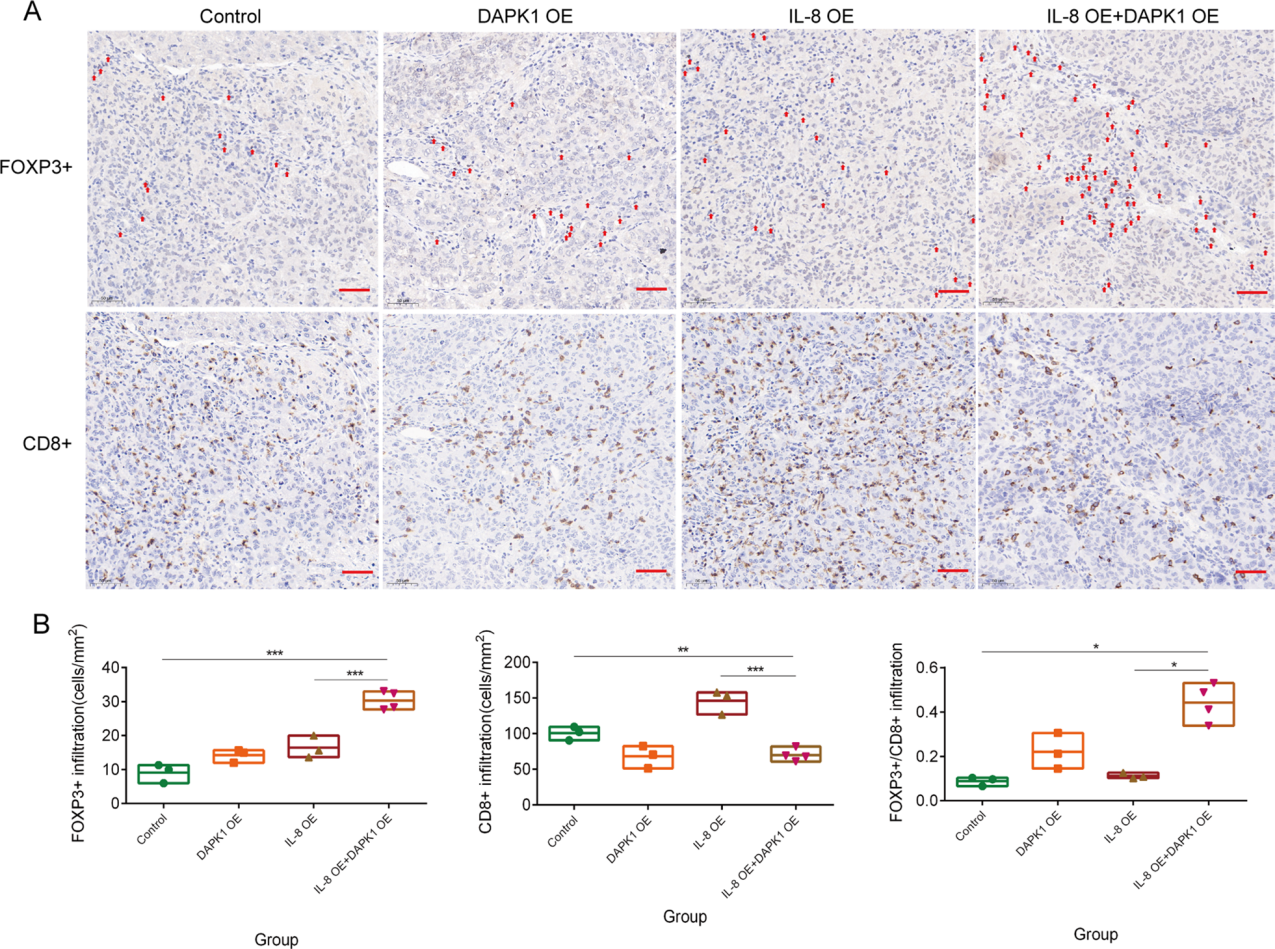
High lactate is the key factor for IL-8 to promote Tregs infiltration in vivo. **A**. Quantification of Tregs (FOXP3 + , red arrow) and CD8 + T cell infiltration in the four mouse orthotopic HCC groups; immunohistochemistry was used to locate the Tregs and CD8 + T cells; scale bar, 50 µm. **B**. The infiltration level of Tregs and CD8 + T cells in HCC tissue of the four mice orthotopic HCC groups (n ≥ 3); Three visual fields were randomly selected for cell counting in each sample. The data represent the mean ± S.D. Mann–Whitney U test or t–tests were used for statistical analysis. *P < 0.05; **P < 0.01; ***P < 0.001. Abbreviations: KD, knockdown; OE, overexpression

Next, we analyzed the infiltration of Tregs and CD8 + T cells in each group (Fig. 8A). Combined with high lactate, high IL-8 expression induced the highest Treg infiltration and the lowest CD8 + expression (Fig. 8B). However, when we removed the high lactate environment, the enhanced Treg polarization was reversed. Therefore, we demonstrated the Treg polarization-promoting effect of lactate and the key role of lactate in transforming IL-8 from proinflammatory to immunosuppressive.

### Entropy model and density checkpoint

**Fig. 9:**
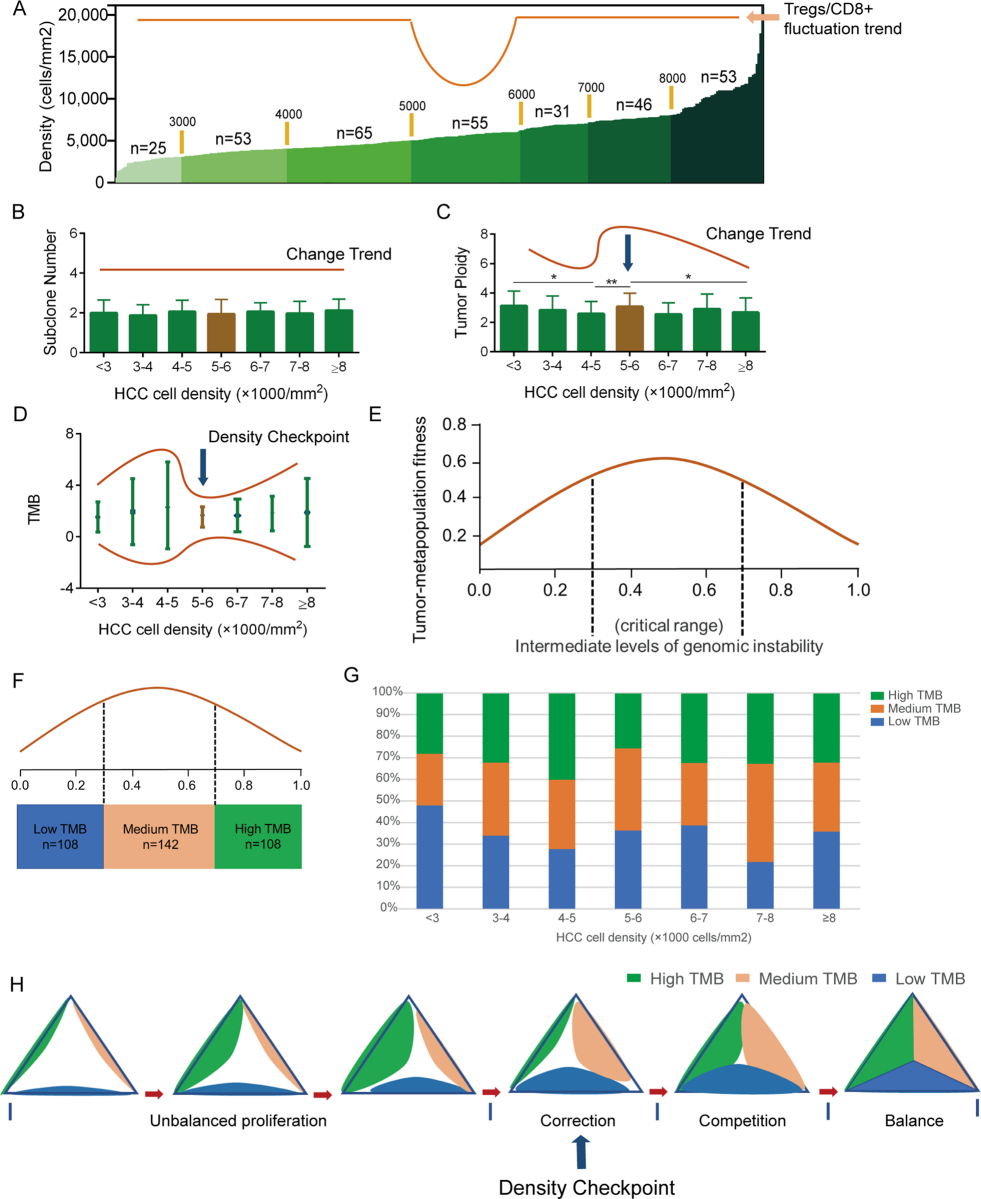
Potential significance of immune fluctuation in HCC. **A**. Grouping method for analyzing variation trend. **B**. The change trend of subclone number with tumor cell density in HCC. **C**. The change trend of HCC ploidy with tumor cell density. **D**. The dispersion tendency of TMB in different groups. **E**. Relationship between tumor-metapopulation fitness and genomic instability (From reference 27). **F**. TMB grouping method following the theory of Hanlee P. Ji et al. **G**. Distribution of TMB in different tumor cell density. **H**. "Entropy model" describes the competition and balance of different TMB groups in the process of tumor development. The data represent the mean ± S.D. Mann–Whitney U test was used for statistical analysis. *P < 0.05; **P < 0.01; ***P < 0.001

In this part, we discuss the potential significance of immune fluctuations based on TCGA database sequencing data combined with tumor cell density. We have described the mode of action of tumor cells on immune cells along the tumor cell density axis and the specific mechanism by which IL-8 promotes Treg infiltration. Immune fluctuation brings about changes in immune pressure to HCC cells, especially enhanced immune pressure with medium cell density (5000–6000 cells/mm^2^) (Fig. 9A). We aimed to determine the effect of enhanced immune pressure on tumor subclones. First, we obtained the number of subclones of HCC samples in TCGA database through CloneSig [24]. The number of subclones did not change with tumor cell density (Fig. 9B). Interestingly, we found that tumor ploidy fluctuated with tumor cell density. In the liver, cell ploidy is closely related to tumor progression. Polyploid restricts the development of HCC through extra copies of tumor suppressor genes and inhibition of proliferation [25]. In densities below 5000 cells/mm^2^, we observed that tumor ploidy decreased with increasing tumor cell density (Fig. 9C). The expansion of low ploidy subclones and a decrease in ploidy caused by proliferation are both possible reasons for this. A decrease in ploidy can dilute the protective effect of the redundant polyploid cell genome and further promote the development of tumor genome instability [26]. In the range of 5000–6000 cells/mm^2^, the decreasing trend in ploidy was reversed. The restoration of polyploidy represents the tendency to inhibit low ploidy subclones and increase the stability of the tumor genome. This phenomenon is consistent with the observed enhanced immune pressure and may be mediated by this immune-modifying effect. However, this brings about further questions. What effect does the reversion of tumor ploidy have on HCC in the process of increasing cell density? Is reversal of tumor ploidy accompanied by convergence of genomic instability? Is it possible that immune fluctuation participates in the modulation of tumor ploidy and genomic instability? What is the significance of immune fluctuation/tumor ploidy modulation/genomic instability on the tumor cell density axis for the development of HCC?

As mentioned above, changes in tumor ploidy will eventually affect the stability of the tumor genome. TMB is a reliable indicator of tumor genomic instability. We used maftools in R to obtain TMB data from the HCC dataset and match it with the tumor cell density. Based on the grouping analysis, we found that the dispersion of TMB increased continuously in densities below 5000 cells/mm^2^, and converged in the range of 5000–6000 cells/mm^2^ (Fig. 9D). The trend in TMB was consistent with that of ploidy. Therefore, the development of HCC genomic instability was controlled in the range of 5000–6000 cells/mm^2^.

Genomic instability is a double-edged sword when it comes to tumor development. It may bring more phenotypes to tumors and push them to their tolerance limit with regards to proliferation [27–29]. Hanlee P. Ji et al. elaborated on the relationship between tumor genomic instability and tumor fitness (Fig. 9E) [27]. In their theory, tumors have a tolerance limit for genomic instability, and the best fitness arises from intermediate levels of genomic instability. The proportion of subclones with high genomic instability needs to be effectively controlled in the tumor population otherwise its advantages will cause tumor collapse under the effect of low dose DNA damage. Here, we used TMB to reflect the degree of tumor genome instability and divided TMB into three groups with Hanlee P. Ji et al.’s theory in mind (Fig. 9F). We identified three TMB groups in different tumor cell densities (Fig. 9G). At a density of less than 5000 cells/mm^2^, the proportion of high TMB consistently increased. Within ranges of 5000–6000 cells/mm^2^, the proportion of high TMB decreased significantly. At a density of > 6000 cells/mm^2^, a perfect balance is achieved between the three TMB groups. The change of immune propensity at 5000–6000 cells/mm^2^ increased immune pressure, which prevented further expansion of the advantages conferred by high TMB subclones and provided opportunities for the remaining subclones to proliferate. One potential mechanism is that immune pressure promotes the genomic instability of some subsets to exceed the tolerance limit; an alternative is that high genomic instability induces the development of a large number of new antigens [30].

The balance between the three TMB subclones provides the best answer for the development of HCC. Based on this, we put forward the “entropy model” (Fig. 9H). We divide this process into four stages, including unbalanced proliferation, correction, competition, and balance. In this process, we regard the density range of 5000–6000 cells/mm^2^ in HCC as the density checkpoint for tumor proliferation (Fig. 9H). Tumors exceeding this checkpoint will likely further proliferate, which in turn may lead to them avoiding collapse caused by the amplification of high TMB.

## Discussion

With the continuous proliferation of tumor subclones, tumor density will increase. During the proliferation of HCC cells, we reported the modes of infiltration of Tregs/CD8 + T cells and M2/M1 macrophages. We first reported on a fluctuation in Treg/CD8 + T cell infiltration. This fluctuation in the Treg/CD8 + T cell ratio with tumor cell density was mainly induced by IL-8 secreted by HCC. IL-8 is a potent inflammatory factor with a strong chemotactic effect on immune cells. The expression of IL-8 in normal tissues is strictly controlled. Therefore, IL-8 is also considered an ideal index for reflecting tumor load and has been shown to indicate poor prognosis [31–33]. IL-8 influences the biology of numerous types of cancer, including melanoma, prostate, colon, pancreatic, liver, breast, and lung cancers [34]. In summary, the tumor-related effects of IL-8 include simultaneous effects on tumor cells and the tumor microenvironment. In targeting tumor cells, IL-8 promotes cell proliferation, epithelial-to-mesenchymal transition, and cell migration [35, 36]. In targeting the tumor microenvironment, IL-8 promotes endothelial cell proliferation, angiogenesis, and recruitment of suppressive immune cell subsets [37, 38]. In the TIME, myelogenous suppressor cells, M2 macrophages, and N2 neutrophils act as the main immunosuppressive cells recruited by IL-8 in tumor tissues [39–41]. The powerful chemotactic effect of IL-8 has been hypothesized to be the key to the recruitment of immunosuppressive cells. Focusing on the recruitment of immunosuppressive cells alone can explain the immunosuppressive state related to IL-8, but the dual chemotactic effect of IL-8 also allows the recruitment of anti-tumor immune cells such as lymphocytes [39, 42, 43]. Relying solely on the recruitment ability of IL-8 may not allow the determination of final immune status nor explain the causes of poor prognosis induction. The reason for the switch to anti-tumor or pro-tumor is a common characteristic of many cytokines in the TIME. In this study, we demonstrated that IL-8 promoted the infiltration of Tregs by promoting lactate production, with lactate as the key factor in this switch. IL-8 promotes lactate production in HCC by promoting the expression of DAPK1 and an increase in PK activity. Due to the different metabolic characteristics of Treg and effector T cells, a high lactate TIME could support the presence of Tregs and damage the function of effector T cells [13]. In addition to Tregs, macrophages, neutrophils, and myelo-derived suppressor cells can respond to the high lactate environment to polarize the direction of immunosuppression and enhance their retention ability [44]. Therefore, in addition to the recruitment ability of IL-8, enhanced lactate production can provide additional explanations for the recruitment of multiple immunosuppressive cells.

The correlation between IL-8 and Treg infiltration increases the potential for IL-8 as a target for HCC immunotherapy. At present, many IL-8-targeted therapies have been developed for the treatment of prostate cancer, non-small cell lung cancer, and HCC [45]. Based on our data, we believe that the key effect of targeting IL-8 in HCC will be to inhibit the establishment of a high lactate environment. A high lactate environment is an obstacle to many treatment methods. Targeted therapy for chemokines with lactate-enhancing effects may mask their potential anti-tumor cell recruitment ability. Blocking the lactate-promoting effect of these chemokines and retaining chemotaxis may be an additional way to enhance the efficacy of treatments. In addition, the immunosuppressive microenvironment is an important feature of solid tumors and a key obstacle to immunotherapy. Owing to the existence of immunosuppressive cells and high lactate concentrations, effector immune cells cannot play a role in the microenvironment [44]. Overcoming this obstacle is an important step in improving the effects of immunotherapy. In this study, we found that the immune microenvironment surrounding HCC is not static. When cell proliferation reaches densities of 5000–6000 cells/mm^2^, the immunosuppressive microenvironment of HCC is reversed. The decline in the Treg/CD8 + T cell ratio in HCC opens a door through the solid immunosuppressive wall. Through this door, it is possible to effectively deliver effector immune cells into the HCC microenvironment. This provides a new way in which to informatively select the immunotherapy window.

Genomic instability provides genetic diversity for tumors. High genomic instability will bring adverse effects to tumors, especially when subsets with high TMB (defective subsets) account for a larger proportion of the total subsets [27, 28]. If defective subsets gain a quantitative advantage, high genomic instability induces tumor to exist in a "high entropy" state and leads to obstacles in tumor survival. Therefore, it is necessary for tumor homeostasis to eliminate defective subsets and constrain mutation burden within an appropriate range before tumor population expansion. Based on immunoediting theory, we propose the “entropy model” and “density checkpoint” to describe the interaction between immunoediting and tumor development in the tumor cell density axis and provide a reasonable explanation for the contradiction between the accumulation of genomic instability and tumor homeostasis. The high TMB purification process is beneficial to the development of HCC and provides a more conducive environment for subsets with an appropriate mutation burden.

## Conclusions

In this study, we identified the mode of infiltration of Tregs/CD8 + T cells on the tumor cell density axis. We found that IL-8 is the mediator of this infiltration and elucidated the mechanisms by which IL-8 promotes Treg infiltration in HCC, providing a new theoretical basis for IL-8 as a therapeutic target for HCC. The decrease in the Treg/CD8 + T cell ratio at densities of 5000–6000 cells/mm^2^ can also inform the selection of immunotherapy windows. Based on tumor ploidy and TMB data, we proposed the “entropy model” and “density checkpoint”, which may provide a reasonable explanation for the contradiction between the accumulation of genomic instability and tumor homeostasis. However, the entropy model and density checkpoint are only based on tumor mutation data and lack experimental validity. The next steps in our research will focus on developing relevant animal models to verify the specific impact of immune fluctuations on the maintenance of tumor homeostasis and exploring the editing role of immune fluctuations. In addition, this phenomenon has only been studied in HCC. We plan to verify the reliability of immune fluctuation and the density checkpoint in more solid tumors and explore the potential mechanism of this phenomenon.

## Supplementary Information

**Additional file 1: Table S1.** Sequences for shRNA induced knockdown. **Table S2.** Antibodies for western blot (WB), immunohistochemistry (IHC), immunofluorescence (IF), and flow cytometry (FC). **Table S3.** Primers for qPCR. **Table S4.** Statistical analysis. **Figure S1.** Influence of basic characteristics of tumor on Treg/CD8+ T cell ratio. **A**. Comparison of Treg/CD8+ T cell ratios in different tumor number groups. **B**. Comparison of Treg/CD8+ T cell ratios in different pathologic grading. **C**. Comparison of Treg/CD8+ T cell ratio in HBsAg+ and HBsAg- group. **D**. Correlation between Tregs/CD8+ T cells ratio and tumor size. The data represent the mean ± S.D. Mann–Whitney U test, Kruskal-Wallis test or spearman analysis were used for statistical analysis. *P < 0.05; **P < 0.01; ***P < 0.001. **Figure S2.** Correlation between the expression of DAPK1 and IL-8 in HCC. **A**. Spearman correlation scatter plot of IL-8 and DAPK1 mRNA expression in HCC; the rho value of the spearman after purity adjustment was used as the degree of correlation. **B**. Expression of DAPK1 in < 5000 cells/mm^2^, 5000–6000 cells/mm^2^ and ≥ 6000 cells/mm^2^ groups; Image J was used for quantification; Scale bar, 20 µm. The data represent the mean ± S.D. Kruskal–Wallis test was used for statistical analysis. *P < 0.05; **P < 0.01; ***P < 0.001.

## Data Availability

The datasets used and/or analysed during the current study are available from the corresponding author on reasonable request.

## Abbreviations

DAPK1: Death-associated protein kinase1
ECAR: Extracellular acidification rate
ELISA: Enzyme-linked immunofluorescence assay
HCC: Hepatocellular carcinoma
HIF1A: Hypoxia inducible factor 1 subunit alpha
HK2: Hexokinase 2
IHC: Immunohistochemistry
PBMCs: Peripheral blood mononuclear cells
PCR: Polymerase chain reaction
PFKFB3: 6-Phosphofructo-2-kinase/fructose-2,6-biphosphatase 3
PK: Pyruvate kinase
TAMs: Tumor-associated macrophages
Tregs: Regulatory T cells
TIME: Tumor immune microenvironment
TMB: Tumor mutation burden
