# Supplementary material for: Tumor cell density dependent IL-8 secretion induces the fluctuation of tregs/CD8 + T cells infiltration in hepatocellular carcinoma: one prompt for the existence of density checkpoint
Source: J Transl Med. 2023 Mar 17;21:202. doi: 10.1186/s12967-023-04060-3 (PMC10022186; doi:10.1186/s12967-023-04060-3)
Supplement: Supplementary file 1 — Additional file 1: Table S1. Sequences for shRNA induced knockdown. Table S2. Antibodies for western blot (WB), immunohistochemistry (IHC), immunofluorescence (IF), and flow cytometry (FC). Table S3. Primers for qPCR. Table S4. Statistical analysis. Figure S1. Influence of basic characteristics of tumor on Treg/CD8+ T cell ratio. A. Comparison of Treg/CD8+ T cell ratios in different tumor number groups. B. Comparison of Treg/CD8+ T cell ratios in different pathologic grading. C. Comparison of Treg/CD8+ T cell ratio in HBsAg+ and HBsAg- group. D. Correlation between Tregs/CD8+ T cells ratio and tumor size. The data represent the mean ± S.D. Mann–Whitney U test, Kruskal-Wallis test or spearman analysis were used for statistical analysis. *P < 0.05; **P < 0.01; ***P < 0.001. Figure S2. Correlation between the expression of DAPK1 and IL-8 in HCC. A. Spearman correlation scatter plot of IL-8 and DAPK1 mRNA expression in HCC; the rho value of the spearman after purity adjustment was used as the degree of correlation. B. Expression of DAPK1 in < 5000 cells/mm2, 5000–6000 cells/mm2 and ≥ 6000 cells/mm2 groups; Image J was used for quantification; Scale bar, 20 µm. The data represent the mean ± S.D. Kruskal–Wallis test was used for statistical analysis. *P < 0.05; **P < 0.01; ***P < 0.001. [file 12967_2023_4060_MOESM1_ESM.docx]

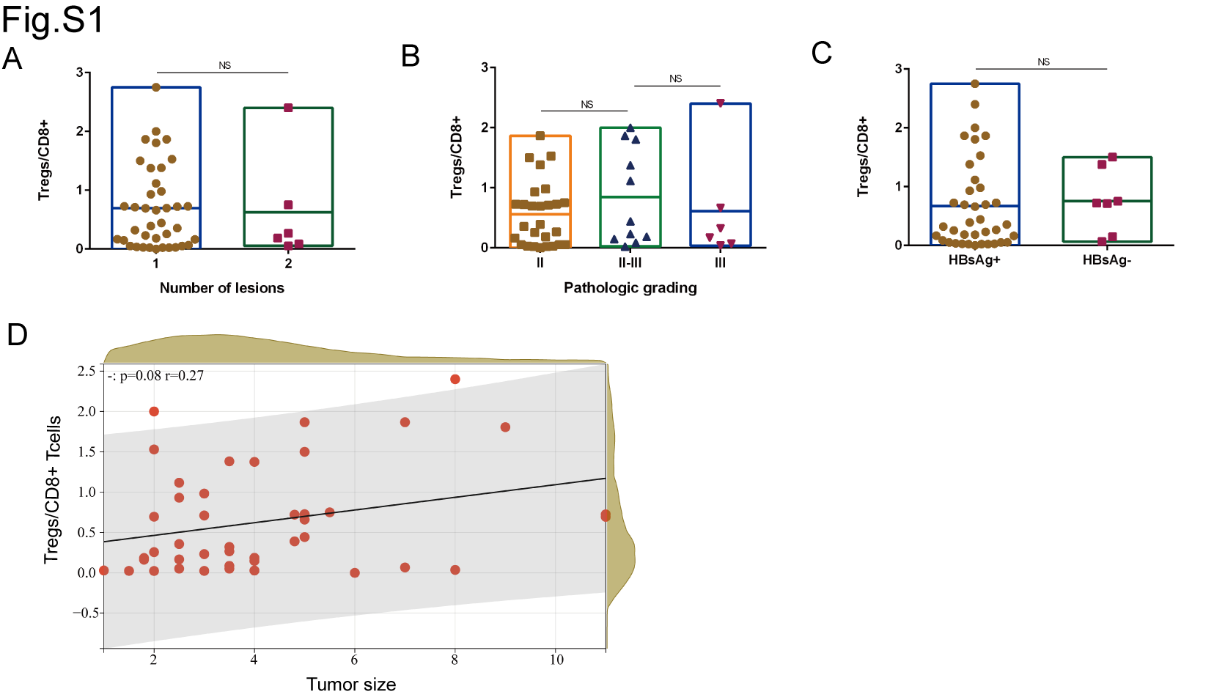


**Figure S1. Influence of basic characteristics of tumor on Treg/CD8+ T cell ratio**

**A.** Comparison of Treg/CD8+ T cell ratios in different tumor number groups. **B**. Comparison of Treg/CD8+ T cell ratios in different pathologic grading. **C**. Comparison of Treg/CD8+ T cell ratio in HBsAg+ and HBsAg- group. **D.** Correlation between Tregs/CD8+ T cells ratio and tumor size. The data represent the mean ± S.D. Mann–Whitney U test, Kruskal-Wallis test or spearman analysis were used for statistical analysis. **P* < 0.05; ***P* < 0.01; ****P* < 0.001.


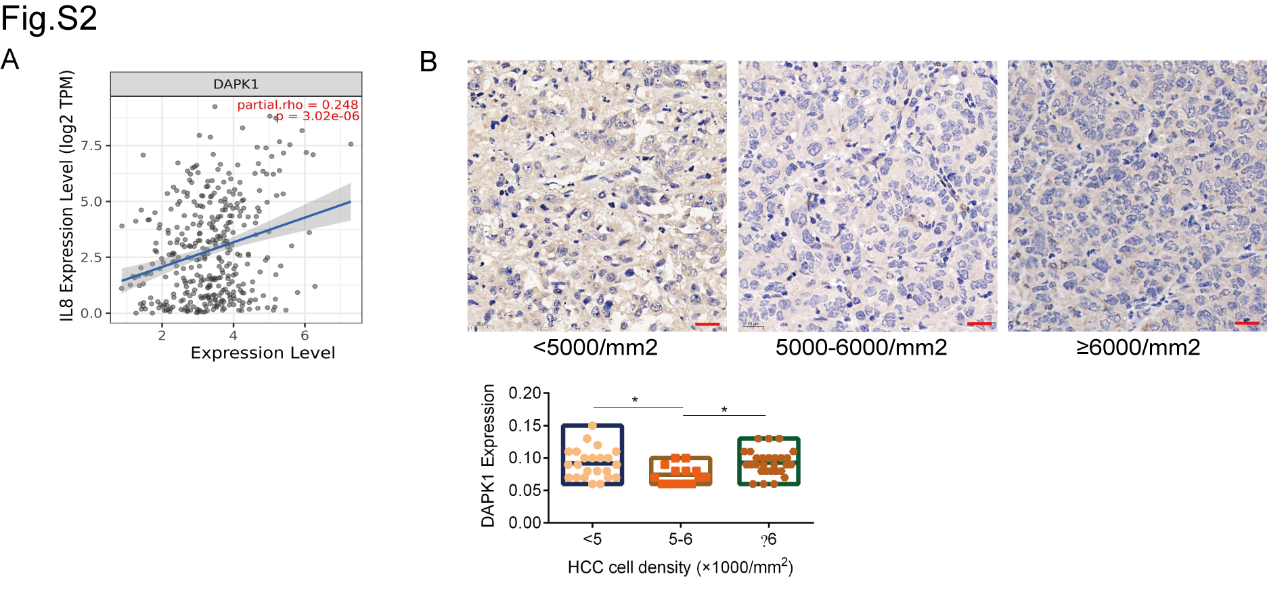


**Figure S2. Correlation between the expression of DAPK1 and IL-8 in HCC**

**A.** Spearman correlation scatter plot of IL-8 and DAPK1 mRNA expression in HCC; the rho value of the spearman after purity adjustment was used as the degree of correlation. **B**. Expression of DAPK1 in < 5000 cells/mm^2^, 5000–6000 cells/mm^2^ and ≥ 6000 cells/mm^2^ groups; Image J was used for quantification; Scale bar, 20 µm. The data represent the mean ± S.D. Kruskal–Wallis test was used for statistical analysis. **P* < 0.05; ***P* < 0.01; ****P* < 0.001.

**Table S1. Sequences for shRNA induced knockdown**

| Name | Sequences |
| --- | --- |
| IL-8 shRNA1 (Human) | CCGGGTGCATAAAGACATACTCCAACTCGAGTTGGAGTATGTCTTTATGCACTTTTTG |
| IL-8 shRNA2 (Human) | CCGGATCAAAGAACTGAGAGTGATTCTCGAGAATCACTCTCAGTTCTTTGATTTTTTG |
| DAPK1 shRNA1 (Human) | CCGGGCTTGATATCACTGTGCCAAACTCGAGTTTGGCACAGTGATATCAAGCTTTTTG |
| DAPK1 shRNA2 (Human) | CCGGCGGATCAAGATCATAGACTTTCTCGAGAAAGTCTATGATCTTGATCCGTTTTTG |

**Table S2. Antibodies for western blot (WB), immunohistochemistry (IHC), immunofluorescence (IF), and flow cytometry (FC)**

| Name | Application | Manufacturer | Catalog No |
| --- | --- | --- | --- |
| Anti-β-Actin antibody | WB | Signalway antibody | 21338 |
| Anti-IL-8 antibody | WB, IHC | R&D system | MAB208 |
| Anti-HIF1A antibody | WB | Signalway antibody | 29547 |
| Anti-PKM2 antibody | WB | Signalway antibody | 21578 |
| Anti-PFKFB3 antibody | WB | Signalway antibody | 49656 |
| Anti-HK2 antibody | WB | Signalway antibody | 32115 |
| Anti-DAPK1 antibody | WB, IHC | proteintech | 25136-1-AP |
| Anti-FOXP3 | IHC | abcam | ab20034 |
| Anti-CD8 | IHC | abcam | ab237710 |
| Anti-CD206 | IHC | abcam | ab64693 |
| Anti-CD86 | IHC | abcam | ab220188 |
| Anti-CD8 alpha antibody | IHC | abcam | ab217344 |
| Anti-FOXP3 antibody | IHC | abcam | ab215206 |
| PE anti-human CD3 | FC | biolegend | 300441 |
| APC anti-human CD25 | FC | biolegend | 302610 |
| FITC anti-human CD4 | FC | biolegend | 317408 |
| PE anti-human FOXP3 | FC | biolegend | 320108 |

**Table S3. Primers for qPCR**

| Name | FOR | REV |
| --- | --- | --- |
| IL-8 | ACTGAGAGTGATTGAGAGTGGAC | AACCCTCTGCACCCAGTTTTC |
| EGF | TCACCTCAGGGAAGATGACC | CAGTTCCCACCACTTCAGGT |
| PDGFA | CAAGACCAGGACGGTCATTT | CCTGACGTATTCCACCTTGG |
| TNF | TCAGAGGGCCTGTACCTCAT | GGAAGACCCCTCCCAGATAG |
| VEGFA | AAGGAGGAGGGCAGAATCAT | ATCTGCATGGTGATGTTGGA |
| VEGFB | CCCTTGACTGTGGAGCTCAT | TCTGCATTCACACTGGCTGT |
| VEGFC | ACCAAACAAGGAGCTGGATG | CTGGGGCAGGTTCTTTTACA |
| TGFB2 | TTGACGTCTCAGCAATGGAG | TCGCCTTCTGCTCTTGTTTT |
| TGFB3 | CTAAGCGGAATGAGCAGAGG | ATTGGGCTGAAAGGTGTGAC |
| IL1A | GTAAGCTATGGCCCACTCCA | AGGTGCTGACCTAGGCTTGA |
| IL1B | GCTGAGGAAGATGCTGGTTC | TCCATATCCTGTCCCTGGAG |
| IL6 | AGGAGACTTGCCTGGTGAAA | CAGGGGTGGTTATTGCATCT |
| IL7 | CTCCCCTGATCCTTGTTCTG | CGAGCAGCACGGAATAAAA |
| IL10 | TGCCTTCAGCAGAGTGAAGA | TGGGTCTTGGTTCTCAGCTT |
| IL11 | GAGCCTGTGGCCAGATACA | AGCTGTAGAGCTCCCAGTGC |
| HIF1A | GAACGTCGAAAAGAAAAGTCTCG | CCTTATCAAGATGCGAACTCACA |
| DAPK1 | ACGTGGATGATTACTACGACACC | TGCTTTTCTCACGGCATTTCT |
| HK2 | TTGACCAGGAGATTGACATGGG | CAACCGCATCAGGACCTCA |
| PFKFB3 | AGCCCGGATTACAAAGACTGC | GGTAGCTGGCTTCATAGCAAC |
| PKM | ATGTCGAAGCCCCATAGTGAA | TGGGTGGTGAATCAATGTCCA |
| β-Actin | CATGTACGTTGCTATCCAGGC | CTCCTTAATGTCACGCACGAT |

**Table S4. Statistical analysis**

| Figure No | Normality test method | *P-*value | Homogeneity test of variance | Statistical method of difference |
| --- | --- | --- | --- | --- |
| Fig.1I left | Kolmogorov-Smirnov | <0.05 | # | Kruskal-Wallis test |
| Fig.1I right | Kolmogorov-Smirnov | >0.05 | >0.05 | One-Way ANOVA |
| Fig.3C | Kolmogorov-Smirnov | <0.05 | # | Kruskal-Wallis test |
| Fig.3D (EGF, IFN, VEGFB, IL11, IL13) | Kolmogorov-Smirnov | <0.05 | # | Kruskal-Wallis test |
| Fig.3D (HGF, PDGFA, TFN, VEGFA, VEGFC, TGFB2, TGFB3, IL1A, IL1B, IL6, IL8, IL7, IL10, IL12A, IL12B) | Kolmogorov-Smirnov | >0.05 | >0.05 | One-Way ANOVA |
| Fig.3F | Kolmogorov-Smirnov | <0.05 | # | Kruskal-Wallis test |
| Fig. 4A-B | Shapiro-Wilk | >0.05 | >0.05 | One-Way ANOVA |
| Fig. 4C | Shapiro-Wilk | >0.05 | >0.05 | One-Way ANOVA |
| Fig. 4C | Shapiro-Wilk | >0.05 | >0.05 | One-Way ANOVA |
| Fig. 4G | Shapiro-Wilk | >0.05 | >0.05 | t-test |
| Fig. 4H left | Shapiro-Wilk | <0.05 | # | Mann-Whitney U test |
| Fig. 4H right | Shapiro-Wilk | >0.05 | >0.05 | t-test |
| Fig. 4I | Shapiro-Wilk | >0.05 | >0.05 | t-test |
| Fig. 5A 5-7 | Shapiro-Wilk | <0.05 | # | Mann-Whitney U test |
| Fig. 5A 8-10 | Shapiro-Wilk | >0.05 | >0.05 | t-test |
| Fig. 5B 5,6,8 | Shapiro-Wilk | >0.05 | >0.05 | t-test |
| Fig. 5B 7,9,10 | Shapiro-Wilk | <0.05 | # | Mann-Whitney U test |
| Fig. 5C 5-6,8-10 | Shapiro-Wilk | >0.05 | >0.05 | t-test |
| Fig. 5C 7 | Shapiro-Wilk | <0.05 | # | Mann-Whitney U test |
| Fig. 5D | Shapiro-Wilk | >0.05 | >0.05 | t-test |
| Fig. 5E | Shapiro-Wilk | >0.05 | >0.05 | t-test |
| Fig. 5F | Shapiro-Wilk | >0.05 | >0.05 | One-Way ANOVA |
| Fig. 5H PF | Shapiro-Wilk | <0.05 | # | Mann-Whitney U test |
| Fig. 5H PF | Shapiro-Wilk | >0.05 | >0.05 | t-test |
| Fig. 5H HI | Shapiro-Wilk | >0.05 | >0.05 | t-test |
| Fig. 5H HK | Shapiro-Wilk | >0.05 | >0.05 | t-test |
| Fig. 6A left | Shapiro-Wilk | >0.05 | >0.05 | t-test |
| Fig. 6A right 5 | Shapiro-Wilk | <0.05 | # | Mann-Whitney U test |
| Fig. 6A right 6-10 | Shapiro-Wilk | >0.05 | >0.05 | t-test |
| Fig. 6B | Shapiro-Wilk | >0.05 | >0.05 | t-test |
| Fig. 6C left | Shapiro-Wilk | >0.05 | <0.05 | Mann-Whitney U test |
| Fig. 6C right | Shapiro-Wilk | >0.05 | >0.05 | t-test |
| Fig. 6E | Shapiro-Wilk | >0.05 | >0.05 | One-Way ANOVA |
| Fig. 6H up | Shapiro-Wilk | >0.05 | >0.05 | t-test |
| Fig. 6H down 5-9 | Shapiro-Wilk | >0.05 | >0.05 | t-test |
| Fig. 6H down 10 | Shapiro-Wilk | <0.05 | # | Mann-Whitney U test |
| Fig. 6I | Shapiro-Wilk | >0.05 | >0.05 | t-test |
| Fig. 6K | Shapiro-Wilk | >0.05 | >0.05 | t-test |
| Fig. 6L left | Shapiro-Wilk | <0.05 | # | Mann-Whitney U test |
| Fig. 6L right | Shapiro-Wilk | >0.05 | >0.05 | t-test |
| Fig. 7A | Shapiro-Wilk | <0.05 | # | Mann-Whitney U test |
| Fig. 7B | Shapiro-Wilk | <0.05 | # | Mann-Whitney U test |
| Fig. 7E | Shapiro-Wilk | >0.05 | >0.05 | t-test |
| Fig. 7F | Shapiro-Wilk | >0.05 | >0.05 | t-test |
| Fig. 8B | Shapiro-Wilk | >0.05 | >0.05 | t-test |
| Fig. 8B F/CD8 | Shapiro-Wilk | <0.05 | # | Mann-Whitney U test |
| Fig. 9C | Kolmogorov-Smirnov | <0.05 | # | Mann-Whitney U test |
| Table 1 (Age, ALB） | Kolmogorov-Smirnov | >0.05 | >0.05 | One-Way ANOVA |
| Table 1（tumor size, TB, ALT, AFP, GGT） | Kolmogorov-Smirnov | <0.05 | # | Kruskal-Wallis test |
| Fig.S1 A, C | Kolmogorov-Smirnov | <0.05 | # | Mann-Whitney U test |
| Fig.S1 B | Kolmogorov-Smirnov | <0.05 | # | Kruskal-Wallis test |
| Fig.S3 B | Kolmogorov-Smirnov | <0.05 | # | Kruskal-Wallis test |
